# Activation of cGMP-Dependent Protein Kinase Stimulates Cardiac ATP-Sensitive Potassium Channels via a ROS/Calmodulin/CaMKII Signaling Cascade

**DOI:** 10.1371/journal.pone.0018191

**Published:** 2011-03-29

**Authors:** Yongping Chai, Dai-Min Zhang, Yu-Fung Lin

**Affiliations:** 1 Departments of Physiology and Membrane Biology, University of California Davis, Davis, California, United States of America; 2 Department of Anesthesiology, University of California Davis, Davis, California, United States of America; Florida International University, United States of America

## Abstract

**Background:**

Cyclic GMP (cGMP)-dependent protein kinase (PKG) is recognized as an important signaling component in diverse cell types. PKG may influence the function of cardiac ATP-sensitive potassium (K_ATP_) channels, an ion channel critical for stress adaptation in the heart; however, the underlying mechanism remains largely unknown. The present study was designed to address this issue.

**Methods and Findings:**

Single-channel recordings of cardiac K_ATP_ channels were performed in both cell-attached and inside-out patch configurations using transfected human embryonic kidney (HEK)293 cells and rabbit ventricular cardiomyocytes. We found that Kir6.2/SUR2A (the cardiac-type K_ATP_) channels were activated by cGMP-selective phosphodiesterase inhibitor zaprinast in a concentration-dependent manner in cell-attached patches obtained from HEK293 cells, an effect mimicked by the membrane-permeable cGMP analog 8-bromo-cGMP whereas abolished by selective PKG inhibitors. Intriguingly, direct application of PKG moderately reduced rather than augmented Kir6.2/SUR2A single-channel currents in excised, inside-out patches. Moreover, PKG stimulation of Kir6.2/SUR2A channels in intact cells was abrogated by ROS/H_2_O_2_ scavenging, antagonism of calmodulin, and blockade of calcium/calmodulin-dependent protein kinase II (CaMKII), respectively. Exogenous H_2_O_2_ also concentration-dependently stimulated Kir6.2/SUR2A channels in intact cells, and its effect was prevented by inhibition of calmodulin or CaMKII. PKG stimulation of K_ATP_ channels was confirmed in intact ventricular cardiomyocytes, which was ROS- and CaMKII-dependent. Kinetically, PKG appeared to stimulate these channels by destabilizing the longest closed state while stabilizing the long open state and facilitating opening transitions.

**Conclusion:**

The present study provides novel evidence that PKG exerts dual regulation of cardiac K_ATP_ channels, including marked stimulation resulting from intracellular signaling mediated by ROS (H_2_O_2_ in particular), calmodulin and CaMKII, alongside of moderate channel suppression likely mediated by direct PKG phosphorylation of the channel or some closely associated proteins. The novel cGMP/PKG/ROS/calmodulin/CaMKII signaling pathway may regulate cardiomyocyte excitability by opening K_ATP_ channels and contribute to cardiac protection against ischemia-reperfusion injury.

## Introduction

The ATP-sensitive potassium (K_ATP_) channel functions as a high-fidelity metabolic sensor which couples intracellular metabolic state to membrane excitability [1]–[3]. The K_ATP_ channel is a hetero-octameric protein [4], [5] composed of four inwardly rectifying potassium channel subunits (Kir6.2 or Kir6.1) [6], [7] and four sulphonylurea receptors (SUR1, SUR2A, or SUR2B) [8], [9]. The molecular compositions of K_ATP_ channels exhibit tissue specificity, which offers substantial diversity across organs. For example, in cardiac and skeletal muscles K_ATP_ channels are composed of Kir6.2 and SUR2A subunits [9], [10], whereas in central neurons and pancreatic β-cells they are composed of Kir6.2 and SUR1 subunits [11]. K_ATP_ channels are widely expressed in excitable tissues and serve a variety of important cellular functions, including glucose-stimulated insulin secretion, neurotransmitter release, vascular tone, and protection of neurons and cardiomyocytes under metabolic stress [12].

K_ATP_ channels are modulated by post-translational mechanisms, such as protein phosphorylation mediated by cAMP-dependent protein kinase (PKA) [13]–[15], Ca^2+^/phospholipid-dependent protein kinase (PKC) [16]–[20], and extracellular signal-regulated kinase (ERK) [21]. The cGMP-dependent protein kinase (PKG), a serine/threonine protein kinase, is increasingly becoming appreciated as an important component of many signal transduction processes in diverse cell types. Functional modulation of K_ATP_ channels by cGMP, presumably through activation of PKG, has been demonstrated in vascular smooth muscle cells [22] and pancreatic β-cells [23]. Our earlier study unravels that PKG bidirectionally regulates the function of neuronal K_ATP_ (*i.e.*, Kir6.2/SUR1) channels, encompassing a predominating stimulatory action, which can be reproduced by nitric oxide (NO) via activation of a cGMP/soluble guanylyl cyclase (sGC)/PKG signaling cascade, and a moderate inhibitory action, which likely involves direct PKG phosphorylation of the channel or some closely associated regulatory protein(s) [24]; more specifically, our findings suggest that the stimulatory action of PKG on neuronal K_ATP_ channels is mediated by intracellular signaling through the 5-hydroxydecanoate (5-HD)-sensitive factor (possibly the mitochondrial K_ATP_ channel), reactive oxygen species (ROS), calcium, and calmodulin [25]. On the other hand, it has been reported that PKG may directly enhance the activity of sarcolemmal K_ATP_ (sarcK_ATP_) channels in cell-free membrane patches obtained from rabbit ventricular cardiomyocytes [26], [27]; however, the underlying mechanism remains unclear.

To elucidate the molecular mechanism responsible for PKG modulation of cardiac K_ATP_ channels, in the present study we employed two model systems: human embryonic kidney (HEK) 293 cells expressing recombinant cardiac-type K_ATP_ channels and acutely isolated ventricular cardiomyocytes containing endogenous sarcK_ATP_ channels. Single-channel recordings of Kir6.2/SUR2A channels, the K_ATP_ channel isoform present in the sarcolemmal membrane of ventricular cardiomyocytes, were performed in both cell-attached and inside-out patch configurations to allow investigation of the functional effect of PKG (activation) on Kir6.2/SUR2A channels as well as the potential involvement of ROS, calmodulin, and calcium/calmodulin-dependent protein kinase II (CaMKII) in signal transduction. We also investigated the role of PKG signaling in modulating the function of sarcK_ATP_ channels in ventricular cardiomyocytes isolated from adult rabbits. Here we demonstrate for the first time that PKG elicits bidirectional regulation of cardiac K_ATP_ channel function, including a predominating stimulatory effect resulting from intracellular signaling, plus a moderate inhibitory effect likely resulting from direct PKG phosphorylation of the channel or some closely associated regulatory proteins. Importantly, our findings provide novel evidence suggesting that PKG stimulates cardiac K_ATP_ channels primarily through generation of ROS, in particular hydrogen peroxide (H_2_O_2_), and subsequently activation of calmodulin and CaMKII, whereas direct PKG phosphorylation of the channel is not involved in channel stimulation. Further, activation of PKG renders changes in the single-channel open and closed properties of cardiac K_ATP_ channels, which may form the kinetic basis of channel stimulation.

## Methods

### Construction of cDNAs

To reconstitute cardiac-type K_ATP_ channels, cDNAs encoding the pore forming subunit Kir6.2 (mouse) and the regulatory subunit SUR2A (rat) were subcloned into mammalian expression vectors pIRES-EGFP (Clontech, Mountain View, CA) and pcDNA3 (Invitrogen, Carlsbad, CA), respectively. The plasmids to be used for transient transfection were prepared with Qiagen maxipreps and verified by DNA sequencing (Qiagen, Valencia, CA).

### Mammalian cell culture and transient transfection

HEK293 cells (ATCC, Manassas, VA) were maintained in Dulbecco's modified Eagle medium DMEM/F12 (Mediatech, Herndon, VA) (supplemented with 2 mM L-glutamine, 10% fetal bovine serum, 100 IU/ml penicillin, and 100 µg/ml streptomycin) at 37°C in humidified 5% CO_2_. Cells were transiently transfected with expression plasmids containing cDNAs of interest using the FuGENE™ 6 reagent (Roche, Indianapolis, IN) in a serum-free medium according to the manufacturer's protocol, or using a modified calcium phosphate-DNA coprecipitation method [28], [29]. Positive transfection was marked by cistronic EGFP expression provided by the vector pIRES-EGFP. The cells were re-plated the following day at a density of 5,000–20,000 cells/dish onto 12 mm glass coverslips pre-coated with 1.5 µg/ml fibronectin (Sigma-Aldrich, St. Louis, MO) to be recorded 48–72 hr after transfection [15].

### Isolation of rabbit ventricular cardiomyocytes

Left ventricular myocytes were isolated from New Zealand White rabbits, using a procedure approved by the Institutional Animal Care and Use Committee of the University of California, Davis (Protocol Number: 13259), in strict accordance with the recommendations in the Guide for the Care and Use of Laboratory Animals of the National Institutes of Health. Rabbits were deeply anesthetized by intravenous injection of pentobarbital sodium (80–100 mg/kg) and all efforts were made to minimize suffering. Hearts were excised, at which time rabbits died insensate by exsanguination. Hearts were quickly placed on a Langendorff apparatus and perfused retrogradely for 5–7 min with nominally Ca^2+^-free Dulbecco's minimum essential medium solution. Then perfusion was switched to the same solution containing 1 mg/ml collagenase with up to 0.1 mg/ml neutral protease. When the heart became flaccid (∼15–30 min), the ventricles were dispersed and filtered. The cell suspension was washed several times in a medium with the Ca^2+^ concentration ([Ca^2+^]) around 150 µM. The cells were subsequently plated on 12 mm glass coverslips coated with laminin to enhance cell adhesion for immediate recordings.

### Electrodes, recording solutions and single-channel recordings

The recording electrodes were pulled from thin-walled borosilicate glass with an internal filament (MTW150F-3; World Precision Instruments, Sarasota, FL) using a P-97 Flaming Brown puller (Sutter Instrument, Novato, CA), and they were then fire-polished to a resistance of 5–10 MΩ. Inside-out and cell-attached single-channel recordings [30] were performed using a recording chamber (RC26; Warner Instruments, Hamden, CT) filled with the intracellular (bath) solution and the recording pipette was filled with the extracellular solution. For HEK293 cells, the intracellular (bath) solution consisted of (in mM): KCl 110, MgCl_2_ 1.44, KOH 30, EGTA 10, HEPES 10, Sucrose 30, pH to 7.2. The extracellular (intrapipette) solution consisted of (in mM): KCl 140, MgCl_2_ 1.2, CaCl_2_ 2.6, HEPES 10, pH to 7.4. For cardiomyocytes, the intracellular (bath) solution consisted of (in mM): KCl 127, MgCl_2_ 1, KOH 13, EGTA 5, HEPES 10, glucose 10, pH to 7.2. The extracellular (intrapipette) solution consisted of (in mM): KCl 140, MgCl_2_ 1, CaCl_2_ 2, HEPES 10, glucose 10, pH to 7.4. In addition, for inside-out recordings, 30 or 100 µM ATP (magnesium salt) was added freshly to the bath recording solution to prevent current rundown and to provide phosphate groups for phosphorylation in the presence of kinases. The use of symmetrical recording solutions (140-mM K^+^) resulted in an equilibrium potential for potassium (E_K_) and a resting membrane potential (Vm) around 0 mV, as determined from the I–V relationship of the K_ATP_ channel. All recordings were carried out at room temperature, and all patches were voltage-clamped at −60 mV (*i.e.*, with +60 mV intrapipette potentials) unless specified otherwise. Single-channel currents were recorded with an Axopatch 200B patch-clamp amplifier (MDS Analytical Technologies-Axon Instruments, Sunnyvale, CA), low-pass filtered (3 dB, 2 kHz), and digitized at 20 kHz on-line using Clampex 9 software (Axon) via a 16-bit A/D converter (Digidata acquisition board 1322A; Axon).

### Preparations of drugs

Working solutions of 8-bromo-cGMP (8-Br-cGMP), 1,4-dihydro-5-(2-propoxyphenyl)-7H-1,2,3-triazolo[4,5-d]pyrimidine-7-one (zaprinast), KT5823, *N*-(2-mercaptopropionyl)glycine (MPG), cGMP, adenosine 5′-triphosphate magnesium salt (MgATP), SKF-7171A, myristoylated autocamtide-2 related inhibitory peptide for CaMKII (mAIP), and pinacidil were diluted from aliquots prior to use with bath recording solutions. Stock solutions were prepared as followed: zaprinast, KT5823, SKF-7171A, and pinacidil in DMSO, and 8-Br-cGMP, cGMP, 5-HD, mAIP, MgATP, and MPG in H_2_O; all were stored at −80°C in aliquots. On the other hand, H_2_O_2_ was prepared fresh daily from the original 30% liquid stock (w/w, approx. 9.8 M). Catalase (human erythrocyte) and PKG Iα (holoenzyme or the catalytic subunit) were diluted from the aliquots of original stocks immediately before use, with corresponding vehicles diluted the same way serving as negative controls. Heat inactivation of PKG was achieved by heating the enzyme at 95°C for at least 15 min before MgATP and cGMP were added to reconstitute the final working solution. All working drug solutions were put on ice and kept away from light. Drugs were applied through a pressure-driven perfusion system (BPS-8; ALA Scientific Instruments, Westbury, NY) to the recording chamber via a micromanifold positioned closely to the patches. The holoenzyme and the catalytic subunit of PKG Iα were obtained from Calbiochem (EMD Biosciences; San Diego, CA); other reagents and chemicals were purchased from Calbiochem or Sigma-Aldrich (St. Louis, MO).

### Data Analysis

Data were analyzed as described before [15], [21], [24], [25], [31], [32]. *Single-channel currents:* Digitized single-channel records of 120-s durations were detected with Fetchan 6.05 (events list) of pCLAMP (Axon) using 50% threshold crossing criterion and analyzed with Intrv5 (Dr. Barry S. Pallotta, University of North Carolina, Chapel Hill, NC; Dr. Janet Fisher, University of South Carolina, Columbia, SC). Analysis was performed at the main conductance level (approximately 70–80 pS). Only patches with infrequent multiple-channel activity were used for single-channel analysis. Duration histograms were constructed as described by Sigworth and Sine [33], and estimates of exponential areas and time constants were obtained using the method of maximal likelihood estimation. The number of exponential functions required to fit the duration distribution was determined by fitting increasing numbers of functions until additional components could not significantly improve the fit [34], [35]. Events with duration less than 1.5 times the system dead time were not included in the fit. Mean durations were corrected for missed events by taking the sum of the relative area (*a*) of each exponential component in the duration frequency histogram multiplied by the time constant (τ) of the corresponding component. Each of the single-channel properties was then normalized to the corresponding controls obtained in individual patches (taken as 1). *Multiple-channel currents:* In patches where multiple-channel activities of K_ATP_ channels were observed for more than 10% of the recording time, the digitized current records were analyzed using Fetchan 6.05 (browse) of pCLAMP to integrate currents in 120-sec segments. The current amplitude (I) values (current amplitude = integrated current/acquisition time) were then normalized to the corresponding controls obtained from the same patches to yield normalized *NPo* (control as 1), as the normalized current amplitude was equivalent to the normalized *NPo* obtained from single-channel analysis when the single-channel conductance remains the same [32]. The normalized *NPo* values obtained from both single-channel and multiple-channel patches were then pooled.

### Statistics

Data are presented as mean ± standard error of the mean (SEM). Statistical comparisons were made using Student's two-tailed one-sample, paired or unpaired *t* tests, or one-way ANOVA followed by Dunnett's multiple comparison tests. Significance was assumed when *P*<0.05. Statistical comparisons were performed using Prism (GraphPad Software, San Diego, CA).

## Results

In the present study, we investigated the molecular mechanism responsible for functional modulation of cardiac K_ATP_ channels elicited by activation of PKG, focusing on two aspects: one is to understand the direct and indirect effects of PKG activation on the function of cardiac K_ATP_ channels, and the other is to elucidate the roles of potential signaling partners of PKG in cardiac K_ATP_ channel modulation. HEK293 cells expressing recombinant Kir6.2/SUR2A (*i.e.*, the cardiac-type K_ATP_) channels and rabbit ventricular cardiomyocytes containing endogenous sarcK_ATP_ channels were employed here as model systems. By performing single-channel recordings in HEK293 cells, we first determined the effect of PKG activators on the activity of Kir6.2/SUR2A channels acquired in the cell-attached patch configuration and that of purified PKG in the inside-out patch configuration, respectively. We subsequently investigated whether ROS, calmodulin, and CaMKII are involved in mediating PKG modulation of Kir6.2/SUR2A channels in intact cells. Further, we compared how exogenous H_2_O_2_ modulates Kir6.2/SUR2A channels in cell-attached versus inside-out patches. We then examined whether H_2_O_2_ modulation of Kir6.2/SUR2A channels in intact cells is dependent on activation of calmodulin and CaMKII. We also investigated the effect of PKG activation and the roles of ROS and CaMKII in mediating PKG's action on sarcK_ATP_ channels in intact rabbit ventricular cardiomyocytes. And lastly, the kinetic changes caused by PKG activation and exogenous H_2_O_2_ on Kir6.2/SUR2A channel opening and closing were delineated.

### Stimulation of Kir6.2/SUR2A channels by PKG activation in intact HEK293 cells

**Figure 1:**
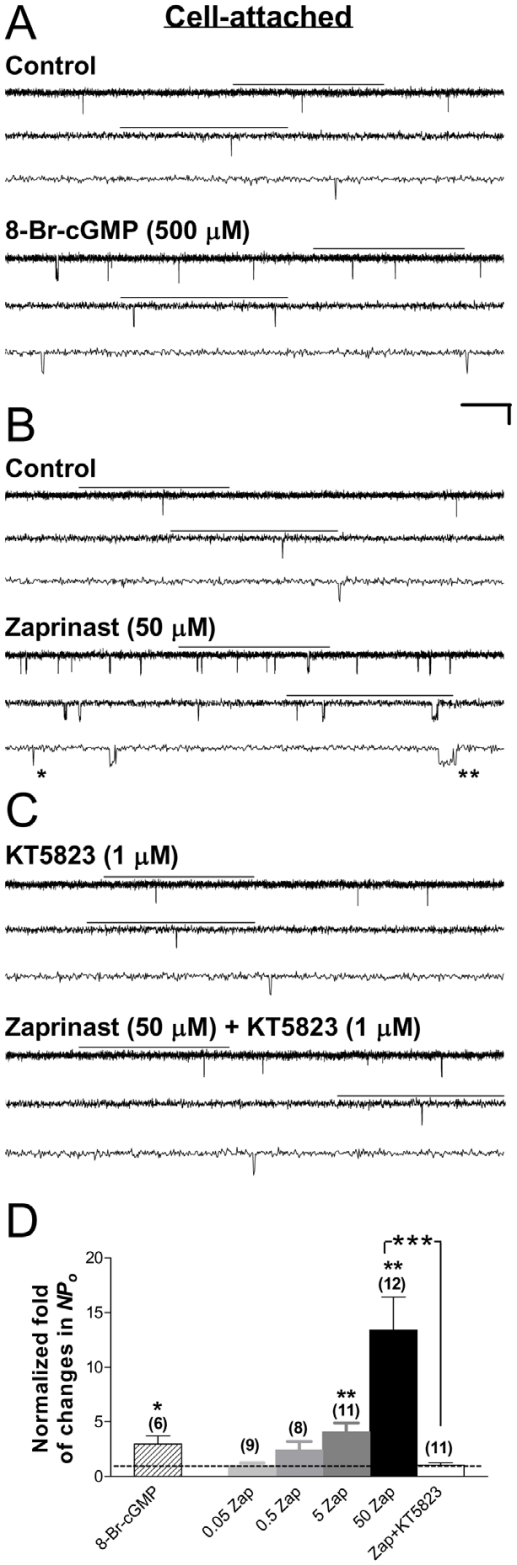
Stimulation of Kir6.2/SUR2A channels by activation of PKG in transfected HEK293 cells. Recombinant Kir6.2/SUR2A channels were expressed in HEK293 cells by transient transfection. Recordings were performed in symmetrical high potassium (140-mM) solutions at room temperature in the cell-attached configuration and the membrane potential was clamped at −60 mV. (A–C) Single-channel current traces of the Kir6.2/SUR2A channel obtained from a representative cell-attached patch prior to (upper panel) and during (lower panel) application of the membrane-permeable cGMP analog 8-Br-cGMP (500 µM) (A), the cGMP-specific PDE inhibitor zaprinast (50 µM) alone (B), or zaprinast (50 µM) plus a selective PKG inhibitor KT5823 (1 µM) following pretreatment with KT5823 for 15 min (C). Downward deflections represent openings from closed states. For all current trace figures, segments of raw recordings marked with a horizontal line on top are shown in successive traces at increasing temporal resolution, revealing singular openings (*) and bursts of openings (**). The horizontal scale bars represent 1 s, 300 ms and 100 ms for traces from top to bottom in each three-trace panel, and the vertical scale bar represents 4 pA. (D) The averaged normalized open probability *NPo* (*i.e.*, relative channel activity) of Kir6.2/SUR2A channels obtained during application of drugs in individual groups of cell-attached patches. Data obtained from patches treated with increasing concentrations of zaprinast (0.05, 0.5, 5 and 50 µM) were also displayed to illustrate the concentration dependence of zaprinast effects. *NPo* values of all groups were normalized to the corresponding control (taken as 1; dashed line) obtained prior to index drug application in individual patches to yield the normalized *NPo*. Data are presented as mean ± SEM of 6–12 patches (number of patches in individual groups provided in parentheses). Significance levels are: *, *P*<0.05; **, *P*<0.01; ***, *P*<0.001 (two-tailed one-sample *t* tests within individual groups, or unpaired *t* tests between groups).

To define the role of PKG in cardiac K_ATP_ channel modulation, we first examined how PKG activation modulates the activity of Kir6.2/SUR2A (*i.e.*, cardiac-type K_ATP_) channels in intact cells. Single-channel currents of Kir6.2/SUR2A channels were recorded in the cell-attached patch configuration to preserve the integrity of the intracellular milieu for potential signaling. To induce PKG activation, 8-bromo-cGMP (8-Br-cGMP), a membrane-permeable cGMP analog, was applied by bath perfusion to intact cells. In Fig. 1 and all other figures illustrating original recording data, segments of current traces marked with a horizontal bar on top are displayed at increasing temporal resolution in successive traces (arranged from top to bottom). The single-channel activity of Kir6.2/SUR2A channels was increased in the presence of 8-Br-cGMP (500 µM) compared with the control currents recorded before drug application from the same cell-attached patch (Fig. 1A), resulting in an increase in the normalized open probability (*NPo*) (control taken as 1) (2.97±0.75; Fig. 1D, hatched bar; 6 patches; *P*<0.05, two-tailed one-sample *t* test). Moreover, zaprinast (50 µM), a membrane-permeable, selective inhibitor of cGMP-specific PDE, also potentiated the single-channel activity of Kir6.2/SUR2A channels in cell-attached patches; the apparent opening frequency and open duration were both higher during zaprinast application (Fig. 1B). To ascertain that the action of zaprinast is specific, we examined whether the effect of zaprinast on the single-channel activity of K_ATP_ channels in intact cells is concentration-dependent. Zaprinast was administered at 0.05, 0.5, 5 or 50 µM by bath application in separate groups of cell-attached patches. We found that zaprinast induced Kir6.2/SUR2A channel activation in a concentration-dependent fashion: there was no effect at 0.05 and 0.5 µM (Fig. 1D, 2nd and 3rd bras from the left; 8–9 patches), whereas at 5 and 50 µM zaprinast increased the normalized *NPo* (*i.e.*, the relative channel activity) of the channel to 4.06±0.85 (Fig. 1D, 4th bar from the left; 11 patches; *P*<0.01, one-sample *t* test) and 13.38±3.05 (Fig. 1D, black filled bar; 12 patches; *P*<0.01), respectively. These results indicate that zaprinast's stimulatory effect on Kir6.2/SUR2A channels results from its specific drug action. In the present study, 50 µM was chosen in all experiments involving the use of zaprinast. In addition, to ensure that the stimulatory effect of PKG activation on the single-channel activity of Kir6.2/SUR2A channels is not biased toward increases due to the low basal activity, the absolute *NPo* (*i.e.*, *NPo* without normalization) values obtained under control and zaprinast-treated condition were also directly compared. Bath perfusion of zaprinast (50 µM) elicited increases in the absolute *NPo* of cardiac-type K_ATP_ channels to different levels in individual cell-attached patches (see Fig. S1, a scatter plot of absolute *NPo* obtained before and during zaprinast treatment); the averaged absolute *NPo* values in the control and zaprinast-treated conditions were 0.04±0.01% and 0.48±0.16%, respectively, manifesting a significant increase by the PKG activator zaprinast (12 data pairs; *P*<0.05, two-tailed paired *t* test). Our hypothesis that PKG activation in intact cells stimulates the function of cardiac-type K_ATP_ channels was supported.

Furthermore, to determine whether zaprinast modulates Kir6.2/SUR2A channel function via activation of PKG, zaprinast (50 µM) was coapplied with the membrane-permeable, selective PKG inhibitor KT5823 (1 µM), following pretreatment with KT5823 (1 µM) for at least 15 min at room temperature. Zaprinast failed to alter the single-channel activity of Kir6.2/SUR2A channels in the presence of the PKG inhibitor KT5823 (Fig. 1C,D, open bar; 11 patches; no significant change), leading to complete abrogation of zaprinast-induced K_ATP_ channel stimulation (Fig. 1D, black filled vs. open bars; *P*<0.01, unpaired t test). The single-channel conductance remained the same. The specificity of KT5823 at 1 µM to selectively inhibit activation of PKG but not that of PKA has been verified in our recent study [25]. Our new data thus indicate that zaprinast stimulated the cardiac-type K_ATP_ channel Kir6.2/SUR2A via activation of a cGMP/PKG mechanism in intact cells. This stimulatory effect of PKG activation on cardiac-type K_ATP_ channels in intact HEK293 cells was reminiscent of a similar effect we observed on neuronal-type K_ATP_ (*i.e.*, Kir6.2/SUR1) channels expressed in two different cell models [24], [32] and, interestingly, appeared to resemble the action of other PKG activators on sarcK_ATP_ channels in intact rabbit ventricular cardiomyocytes [26], [27].

### Reduction in Kir6.2/SUR2A single-channel activity by direct application of PKG in inside-out patches excised from HEK293 cells

**Figure 2 pone-0018191-g002:**
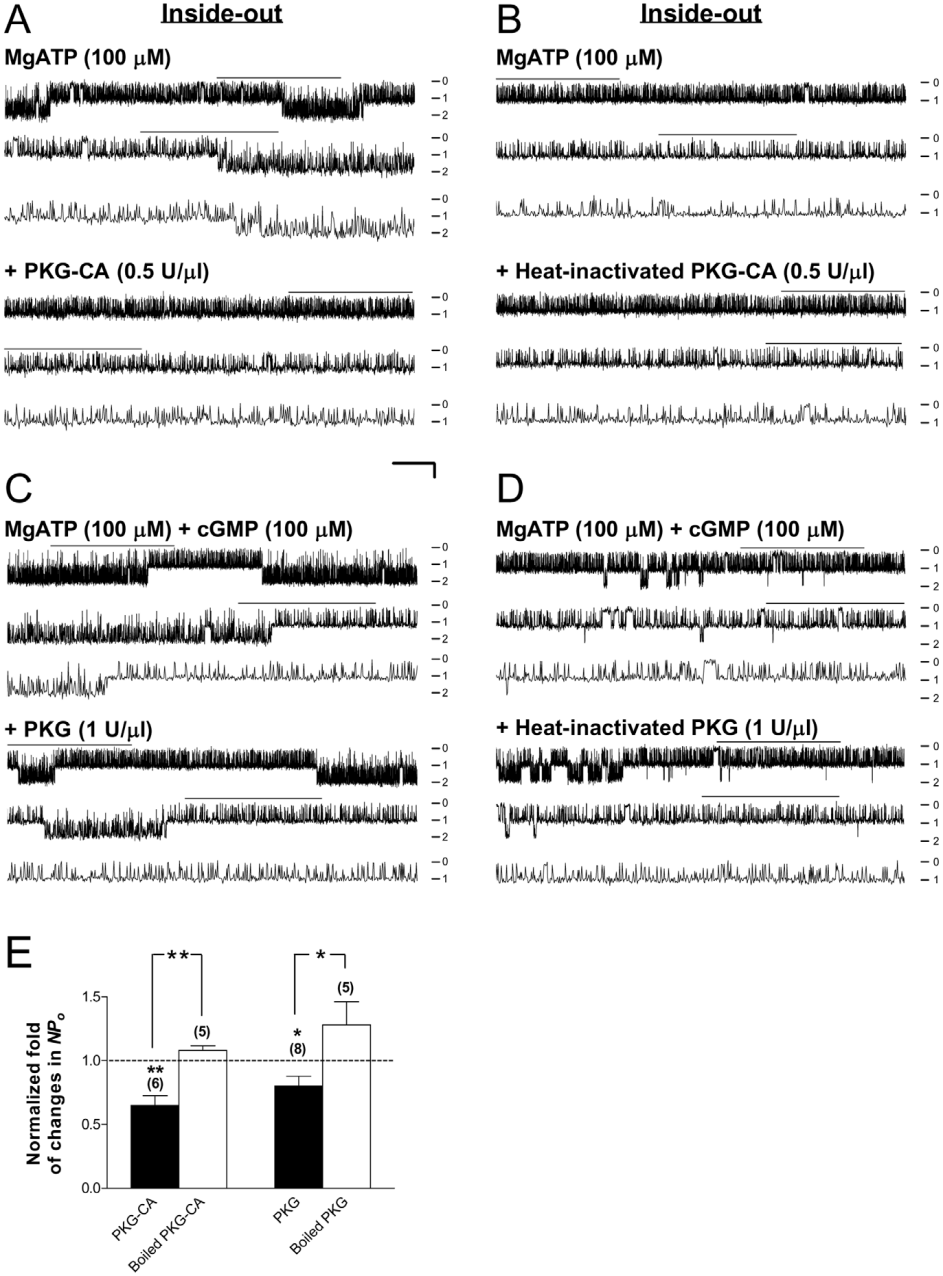
Suppression of Kir6.2/SUR2A channel activity by direct application of purified PKG in excised inside-out patches. Single-channel currents were obtained in the inside-out patch configuration from transiently transfected HEK293 cells. Recordings were conducted in symmetrical high potassium (140-mM) solutions and the membrane potential was clamped at −60 mV. (A–D) Single-channel current traces of Kir6.2/SUR2A channels in a representative inside-out patch prior to (upper panel) and during (lower panel) bath perfusion of the catalytic subunit of PKG (PKG-CA; 0.5 U/µl) (A), heat-inactivated PKG-CA (B), purified PKG Iα holoenzyme (PKG; 0.5 U/µl) (C), or heat-inactivated PKG holoenzyme (D). Numbers (from 0–2) marked at the right margin of current traces indicate the level of simultaneous channel opening: 0 for closed-channel, 1 for one-channel, and 2 for two-channel level opening. Scale bars are the same as described in Fig. 1. The single-channel currents of K_ATP_ channels in inside-out patches were usually higher than in cell-attached patches (Figs. 2A vs. 1A), as ATP inhibition of the channel was partially alleviated upon patch excision. (E) Normalized *NPo* values of Kir6.2/SUR2A channels obtained during applications of live or heat-inactivated PKG in individual groups of inside-out patches. The drug effect was compared with the corresponding control obtained from the same patch and *NPo* values were normalized as described in Fig. 1D (control taken as 1; dashed line). The average data are presented as mean ± SEM of 5–8 patches. Significance level is: *, *P*<0.05; **, *P*<0.01 (two-tailed one-sample *t* tests within individual groups, or unpaired *t* tests between groups).

Does stimulation of the Kir6.2/SUR2A, the cardiac-type K_ATP_, channel by activation of PKG in intact cells (see Fig. 1) result from direct PKG phosphorylation of the channel, or alternatively, involve phosphorylation of some intermediary, regulatory protein that in turn activates the channel? To distinguish between these two possibilities, we applied the purified catalytic subunit of PKG Iα (*i.e.*, PKG-CA) directly to the cytoplasmic surface of inside-out patches excised from transfected HEK293 cells that expressed Kir6.2/SUR2A channels. One advantage of using PKG-CA is that it allows examination of PKG-specific effects in the absence of the PKG coactivator cGMP, thereby lessoning concerns about potential cGMP-induced, exogenous PKG-independent effect on the channel during inside-out recordings. The single-channel activity of K_ATP_ channels was higher in inside-out patches than in cell-attached patches (Figs. 2 vs. 1), which was as expected due to partial relief of ATP inhibition upon patch excision. Single-channel currents of Kir6.2/SUR2A channels in inside-out patches were continuously acquired before and during bath perfusion of solutions containing the following: MgATP (100 µM) for a duration of 6 min or until the channel activity was stabilized, and subsequently PKG-CA (0.5 U/µl) plus MgATP (100 µM) for at least 10 min. The *NPo* obtained during coappliation of PKG-CA and MgATP was normalized to the corresponding MgATP control obtained in individual inside-out patches for statistical comparisons. Intriguingly, PKG-CA reduced, rather than enhanced, Kir6.2/SUR2A single-channel activity recorded in the inside-out patch configuration (Fig. 2A), while the heat-inactivated PKG-CA administered in lieu of the live enzyme elicited no change (Fig. 2B); the normalized *NPo* values were 0.65±0.08 (control taken as 1) (Fig. 2E, 1st bar from the left; 6 patches; *P*<0.01, one-sample *t* test) and 1.08±0.04 (Fig. 2E, 2nd bar from the left; 5 patches; no significant change), respectively. The single-channel conductance remained unchanged. Hence, the inhibitory effect of PKG-CA on Kir6.2/SUR2A channels was significantly abolished by heat inactivation of the enzyme (Fig. 2E, 1st vs. 2nd bars; *P*<0.01, two-tailed unpaired *t* test). These results indicate that PKG-CA exerted a specific, inhibitory action on the function of cardiac-type K_ATP_ channels expressed in HEK293 cells, which became evident only in cell-free patches (Fig. 2A,B,E) but not in intact cells.

Considering that PKG-CA has a smaller molecular weight compared with the holoenzyme and therefore the accessibility (to targets) may or may not be the same, we proceeded to verify the effect of PKG-CA on Kir6.2/SUR2A channels using purified PKG Iα holoenzyme (PKG). The single-channel activity of these channels in inside-out membrane patches excised from transfected HEK293 cells was monitored in the continuous presence of MgATP (100 µM) before and during sequential addition of cGMP (100 µM; for at least 6 min) and the PKG holoenzyme (1 U/µl; for at least 10 min) to the cytoplasmic surface of patches. The *NPo* obtained during application of the PKG holoenzyme mix was normalized to the corresponding cGMP/MgATP control obtained before enzyme application from the same patches. Similar to PKG-CA, PKG holoenzyme reduced the apparent opening frequency of Kir6.2/SUR2A channels acquired in the inside-out patch configuration (Fig. 2C), while the single-channel conductance remained the same; the averaged normalized *NPo* was 0.80±0.08 (control taken as 1) (Fig. 2E, 3rd bar from the left; 8 patches; *P*<0.05, one-sample *t* test). By contrast, the heat-inactivated PKG was incapable of suppressing the activity of Kir6.2/SUR2A channels (Fig. 2D); the normalized *NPo* obtained from a group of five patches was 1.28±0.18 (Fig. 2E, 4th bar; no significant change). The significant abolishment of the inhibitory effect of PKG holoenzyme by heat inactivation (Fig. 2E, 3rd vs. 4th bars; *P*<0.05, unpaired *t* test) once again indicates that that the inhibitory action of PKG was specific. Our findings obtained from both PKG-CA and PKG holoenzyme groups thus imply that in addition to being stimulated by PKG (activation) in intact cells (see Fig. 1), the Kir6.2/SUR2A channel was also subject to moderate inhibition mediated by PKG-mediated phosphorylation, an effect unmasked in the cell-free condition when intracellular signaling was prevented.

### Effects of ROS scavenging on PKG stimulation of Kir6.2/SUR2A channels in intact HEK293 cells

**Figure 3 pone-0018191-g003:**
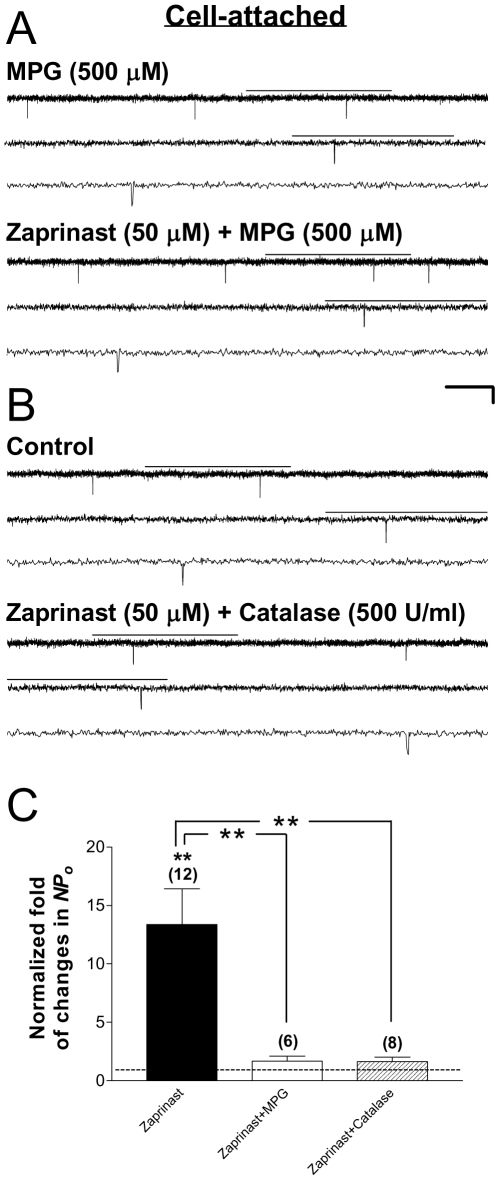
The role of ROS, particularly H_2_O_2_, in mediating Kir6.2/SUR2A channel stimulation downstream of PKG activation. Single-channel currents were obtained from cell-attached patches obtained from transiently transfected HEK293 cells. Recordings and drug application were administered as described in Fig. 1. (A–B) Single-channel current traces of the Kir6.2/SUR2A channel obtained from a cell-attached patch prior to (upper panel) and during (lower panel) application of the cGMP-specific PDE inhibitor zaprinast (50 µM) together with a membrane-permeable ROS scavenger MPG (500 µM) after 15-min preincubation in MPG (A), or with catalase, a H_2_O_2_-decomposing enzyme (500 U/ml) (B). There was no pretreatment for cells in the catalase group because catalase is not membrane-permeable. Scale bars are the same as described in Fig. 1. (C) Normalized *NPo* values of Kir6.2/SUR2A channels obtained during applications in individual groups of cell-attached patches. *NPo* values were normalized to the corresponding control as described in Fig. 1D (control taken as 1; dashed line). The zaprinast data (filled bar) are the same as presented in Fig. 1D, and are included here for comparison purpose. Data are presented as mean ± SEM of 6–12 patches. The significance level is: **, *P*<0.01 (two-tailed one-sample *t* tests within individual groups, or Dunnett's multiple comparison tests between groups).

Our results described thus far indicate that PKG exerted dual functional regulation on cardiac-type K_ATP_ channels, with a predominating stimulatory effect preserved in intact cells (see Fig. 1) and a relatively mild inhibitory effect evident only in cell-free patches (see Fig. 2), and therefore it is conceivable that an indirect signaling mechanism rather than direct PKG phosphorylation of the channel may be responsible for K_ATP_ channel stimulation by PKG. PKG activation has been demonstrated to account for NO-induced ROS generation in rat cardiomyocytes as well as the anti-infarct effect of NO in intact, isolated heart [36], which suggests that ROS generation may be induced by PKG activation in cardiac tissues. Interestingly, our recent findings also suggest that ROS are required for the acute, stimulatory action of PKG on neuronal-type K_ATP_ channels [25]. Would ROS serve as intermediate signals inducible upon activation of PKG to mediate an acute effect of the enzyme on cardiac-type K_ATP_ channels? To answer this question, we examined the effect of *N*-(2-mercaptopropionyl)glycine (MPG), an ROS scavenger, on PKG stimulation of Kir6.2/SUR2A channels in intact HEK293 cells. Following pretreatment with MPG (500 µM) for at least 15 min at room temperature, subsequent coapplication of the selective cGMP-specific PDE inhibitor zaprinast (50 µM) and MPG (500 µM) via bath perfusion did not alter the single-channel currents of Kir6.2/SUR2A channels in cell-attached patches (Fig. 3A). The averaged, normalized *NPo* obtained during coapplication of zaprinast and MPG was 1.68±0.42 (control as 1) (Fig. 3C, open bar; 6 patches; no significant change, one-sample *t* test), which was significantly reduced from the marked increase obtained from patches treated with zaprinast alone (Fig. 3C, filled vs. open bars; *P*<0.01, Dunnett's multiple comparison test following one-way ANOVA). These data thus indicate that ROS were indispensible signals for PKG stimulation of cardiac-type K_ATP_ channels in intact cells.

### Effects of catalase, the H_2_O_2_ decomposing enzyme, on Kir6.2/SUR2A channel stimulation induced by PKG activation in intact HEK293 cells

Among ROS, H_2_O_2_ is a relatively stable form. The potential involvement of H_2_O_2_ in mediating the stimulatory effect of PKG on cardiac-type K_ATP_ channels was examined by coapplying catalase, an enzyme that decomposes H_2_O_2_ to water and oxygen, together with zaprinast to cell-attached patches obtained from transfected HEK293 cells that expressed Kir6.2/SUR2A channels. We found that in the presence of catalase (500 U/ml), zaprinast (50 µM) was incapable of activating Kir6.2/SUR2A channels in individual cell-attached patches (Fig. 3B); the averaged normalized *NPo* was 1.64±0.38 (control as 1) (Fig. 3C, hatched bar; 8 patches; no significant change, one-sample *t* test), which was in sharp contrast to the significant increase in patches receiving only zaprinast treatment (Fig. 3C, filled vs. hatched bars; *P*<0.01, Dunnett's multiple comparison test). These results indicate that removal of H_2_O_2_ and related ROS prevented zaprinast from exerting its stimulatory action on Kir6.2/SUR2A channels in intact HEK293 cells, which supports our working model that ROS (and especially H_2_O_2_) play a crucial role in mediating PKG stimulation of cardiac K_ATP_ channels.

### Suppression of calmodulin activity abolished Kir6.2/SUR2A channel stimulation by activation of PKG in intact HEK293 cells

**Figure 4 pone-0018191-g004:**
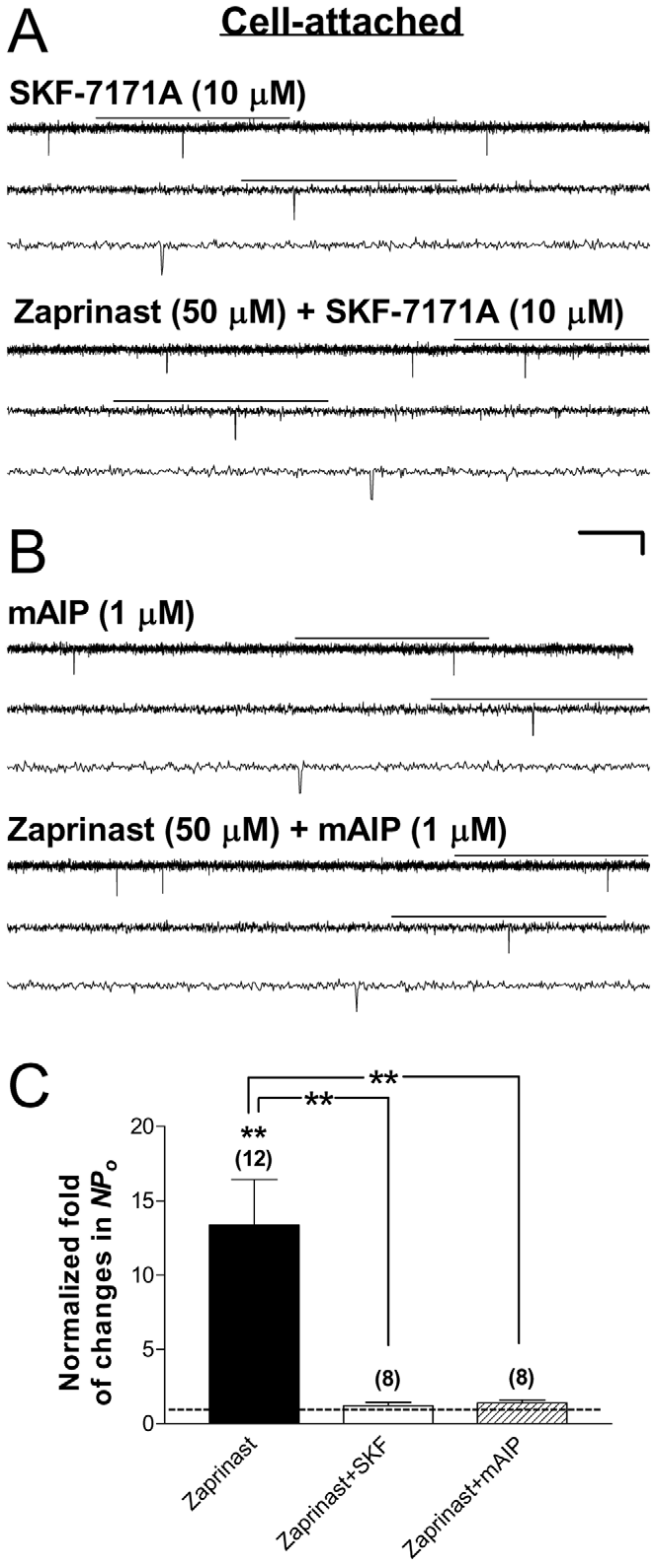
Roles of calmodulin and CaMKII in mediating PKG stimulation of Kir6.2/SUR2A channels. Recombinant Kir6.2/SUR2A channels were expressed in HEK293 cells by transient transfection. Cell-attached patch-clamp recordings and drug application were administered as described in Fig. 1. (A–B) Single-channel current traces of Kir6.2/SUR2A channel obtained from a cell-attached patch prior to (upper panel) and during (lower panel) application of the cGMP-specific PDE inhibitor zaprinast (50 µM) together with the membrane-permeable, selective calmodulin antagonist SKF-7171A (10 µM) (A), or with the myristoylated autocamtide-2 related inhibitory peptide for CaMKII (mAIP; 1 µM) (B). The scale bars are the same as described in Fig. 1. (C) Normalized *NPo* of Kir6.2/SUR2A channels obtained during application of drugs in individual groups. The zaprinast data (filled bar) are the same as presented in Fig. 1D, and are included here for comparison purpose. *NPo* values were normalized to the corresponding control (taken as 1) as described in Fig. 1D. The dashed line indicates the control level. Data are presented as mean ± SEM of 8–12 patches. The significance level is: **, *P*<0.01 (two-tailed one-sample *t* tests within individual groups, or Dunnett's multiple comparison tests between groups).

Our recent study suggests that calmodulin mediates PKG stimulation of neuronal K_ATP_ channels in intact cells [25]. Whether calmodulin is required for the functional effect of PKG activation on cardiac-type K_ATP_ channels is not known. To examine this possibility, SKF-7171A (10 µM), a cell-permeable calmodulin antagonist, was coapplied together with the PKG activator zaprinast (50 µM) to Kir6.2/SUR2A channels in cell-attached patches obtained from transfected HEK293 cells, following a 15-min pretreatment with SKF-7171A (10 µM). In the presence of SKF-7171A, zaprinast failed to increase the single-channel activity of Kir6.2/SUR2A channels in intact cells (Fig. 4A), resulting in an averaged, normalized *NPo* of 1.23±0.23 (control as 1) (Fig. 4C, open bar; 8 patches; no significant change, one-sample *t* test). The positive zaprinast effect on the normalized *NPo* of Kir6.2/SUR2A channels was completely abolished by SKF-7171A (Fig. 4C, filled vs. open bars; *P*<0.01, Dunnett's multiple comparison test following one-way ANOVA), indicating that the activity of calmodulin was required for PKG stimulation of cardiac-type K_ATP_ channels in intact cells.

### Inhibition of CaMKII prevented PKG stimulation of Kir6.2/SUR2A channel function in intact HEK293 cells

CaMKII is one of the major regulators of Ca^2+^ homeostasis in the heart, phosphorylating cardiac contractile regulatory proteins. CaMKII has been shown to affect the function of cardiac ion channels [37]–[39]. Would CaMKII be part of the signal transduction mechanism responsible for K_ATP_ channel stimulation following activation of PKG and calmodulin? To determine whether CaMKII activation mediates the stimulatory effect of PKG on cardiac-type K_ATP_ channels, we pretreated cells with the cell-permeable, myristoylated autocamtide-2 related inhibitory peptide selective for CaMKII (mAIP; 1 µM) for at least 15 min at room temperature, followed by coapplying the PKG activator zaprinast (50 µM) plus mAIP (1 µM) during continuous cell-attached patch recordings of Kir6.2/SUR2A channel currents. We found that activation of PKG by zaprinast no longer enhanced the single-channel activity of Kir6.2/SUR2A channels in the presence of mAIP (Fig. 4B); the averaged normalized *NPo* was 1.40±0.21 (Fig. 4C, hatched bar; 8 patches; no significant change), which represented a significant blockade of PKG's stimulatory effect (Fig. 4C, filled vs. hatched open bars; *P*<0.01, Dunnett's multiple comparison test following one-way ANOVA). These results indicate that PKG stimulation of cardiac-type K_ATP_ channels was dependent on the activity of CaMKII in intact cells.

### Kinetic effects on Kir6.2/SUR2A channel opening and closing exerted by activation of PKG in intact HEK293 cells

Channel function and its modulation is determined by the conformational changes that the channel undertakes to enable opening or closure of the pore for ion permeation, as reflected by the number of open and closed states it exhibits and the rates of transitions between different states. To understand the effect rendered by PKG activation on cardiac-type K_ATP_ channel gating, we investigated whether zaprinast, by triggering cGMP/PKG signaling, causes more frequent entry into the open state (*i.e.*, increases the opening frequency), prolongs stay in the open state (*i.e.*, increases the open duration/time constant of certain open state), decreases dwelling time in the closed states (*i.e.*, decreases the closed duration/time constant of certain closed state), stabilizes or destabilizes the occurrence of a particular state (*i.e.*, shifts the relative distribution among states), or induces any combination of the above.

**Figure 5 pone-0018191-g005:**
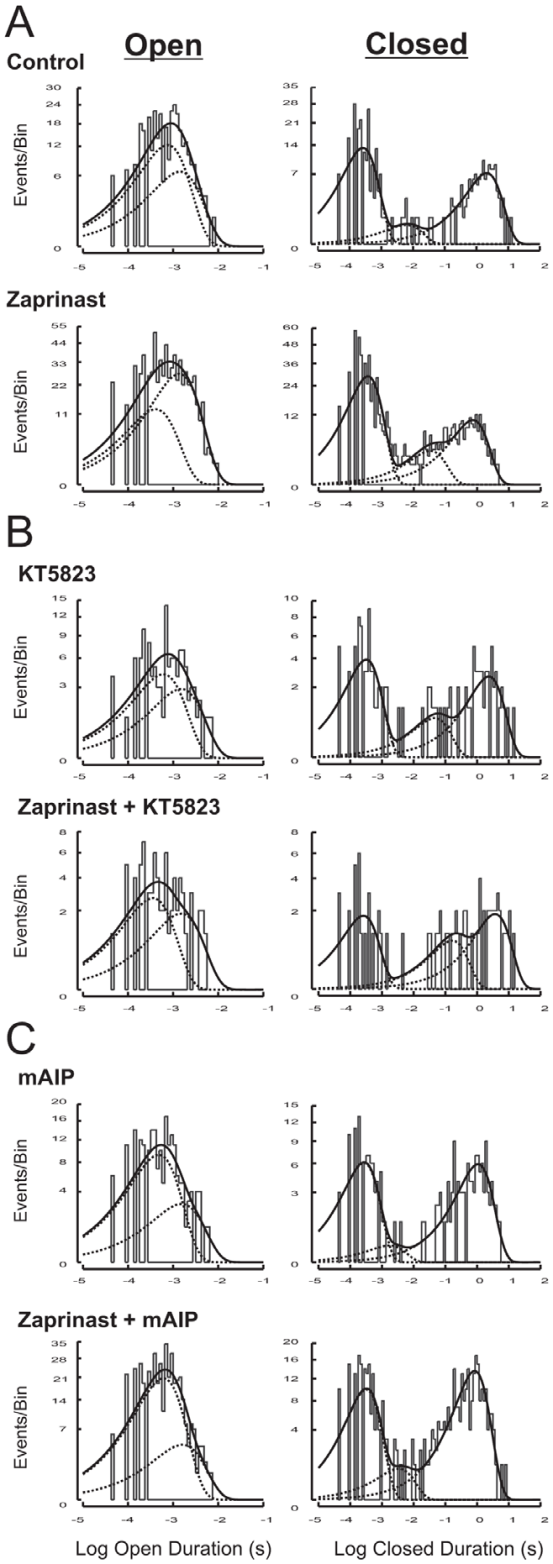
Effects of PKG activation on open- and closed-duration distributions of Kir6.2/SUR2A channels in intact cells. Data were obtained from transfected HEK293 cells expressing Kir6.2/SUR2A channels. Frequency histograms of duration distributions fitted from events obtained before (upper panel) and during (lower panel) the application of zaprinast (50 µM) alone (A), zaprinast (50 µM) plus a selective PKG inhibitor KT5823 (1 µM) (B), or zaprinast (50 µM) plus the myristoylated CaMKII inhibitory peptide mAIP (1 µM) (C) in representative cell-attached patches. Cells were incubated with respective inhibitors for at least 15 min before index drug perfusion. Frequency histograms displayed are open-duration distribution (left column) and closed-duration distributions (right column), respectively, for all patches. Duration histograms were constructed as described in Methods.

Single-channel analysis of Kir6.2/SUR2A channel currents in cell-attached patches obtained from HEK293 cells before and during zaprinast treatment was conducted. The fitting results revealed that open- and closed-duration distributions of the Kir6.2/SUR2A channel could be best described by a sum of two open components and a sum of three closed components, respectively (Fig. 5A; a representative patch). During zaprinast application, the Kir6.2/SUR2A channel exhibited a tendency to open into the longer open state more frequently in comparison with its opening pattern under the control condition (Fig. 5A, Open; *top panel:* control; *bottom panel:* zaprinast); the relative area of the longer open component (designated as O_2_) was increased while that of the shorter open state (designated as O_1_) was reduced in the same patch (Fig. 5, Open), which led to a small yet significant increase in the normalized corrected mean open duration during zaprinast treatment in individual patches (Table 1, Zaprinast; 7 patches; *P*<0.05, two-tailed one-sample *t* test). Besides an increase in the corrected mean open duration, the opening frequency was also increased by zaprinast (Table 1, Zaprinast; *P*<0.05), and the combined effect was an elevated normalized *NPo* (*P*<0.05). With regard to the closed duration distributions, during zaprinast treatment the Kir6.2/SUR2A channel exhibited an increase in the occurrence of closures at the shorter closed states while reducing the occurrence of closures at the longest closed state (*i.e.*, to shift the relative area under the longest closed component toward the shorter ones); moreover, the time constant (which depicts the dwelling time) of the longest closed state (designated as C_3_) was reduced compared with the corresponding control (Fig. 5A; Closed; *top panel:* control; *bottom panel:* zaprinast). These changes in the channel closing pattern largely accounted for a decrease in the normalized mean closed duration observed during zaprinast treatment (Table 1, Zaprinast; *P*<0.0001, one-sample *t* test). Importantly, we found that the changes induced by zaprinast on the relative distribution of open and closed states as well as other single-channel properties were prevented when the PKG inhibitor KT5823 (1 µM) was coapplied (Fig. 5B, a representative cell-attached patch; Table 1, KT5823; 9 patches), indicating that the aforementioned effects of zaprinast on cardiac-type K_ATP_ channel gating were mediated by activation of PKG.

**Table 1:** Roles of ROS, calmodulin and CaMKII in mediating the stimulatory effects of PKG on the normalized single-channel open and closed properties of recombinant Kir6.2/SUR2A channels expressed in intact HEK293 cells.

Properties	Zaprinast	+KT5823	+MPG	+Catalase	+SKF-7171A	+mAIP
Open probability	13.75±4.91*	1.13±0.26	1.61±0.51	1.49±0.48	1.12±0.23	1.40±0.21
Opening frequency	8.84±2.51*	1.05±0.20	1.27±0.26	1.38±0.45	0.92±0.17	1.31±0.14
Mean open duration	1.47±0.17*	1.04±0.07	1.13±0.12	1.17±0.13	1.17±0.18	1.07±0.09
Mean closed duration	0.16±0.04****	1.10±0.18	0.88±0.15	0.79±0.17	1.87±0.39	0.96±0.16
Number of patches	7	9	5	5	7	8

Single-channel recordings of Kir6.2/SUR2A channels in cell-attached patches obtained from transfected HEK293 cells were performed at −60 mV in symmetrical 140-mM K^+^ solutions. Zaprinast (50 µM), or zaprinast plus KT5823 (1 µM), MPG (500 µM), catalase (500 U/ml), SKF-7171 (10 µM), or mAIP (1 µM), was applied by bath perfusion using a pressure-driven system. The single-channel properties were obtained as described in Methods. All values were normalized to the corresponding controls obtained in individual patches prior to index drug application (control taken as 1), averaged and are presented as mean ± SEM. Significance levels are:

*, *P*<0.05;

****, *P*<0.0001 (two-tailed one-sample *t* tests).

### Changes in the single-channel open and closed properties of Kir6.2/SUR2A channels by activation of PKG exhibited dependence on the activities of ROS, calmodulin, and CaMKII

To delineate the roles played by ROS, H_2_O_2_, calmodulin and CaMKII in mediating the kinetic effects of PKG activation on cardiac-type K_ATP_ channels, we analyzed the single-channel properties of Kir6.2/SUR2A channels expressed in transfected HEK293 cells, including *NPo*, opening frequency, corrected mean open duration and mean closed duration, in patches suitable for single-channel analysis (see Methods for details). Changes in the single-channel properties of Kir6.2/SUR2A channels in intact HEK293 cells caused by the PKG activator zaprinast (Table 1, Zaprinast; one-sample *t* test), including the reduction in the mean closed duration and increases in the opening frequency and the corrected mean open duration (which led to an increase in the normalized *NPo*), were ablated not only by the PKG inhibitor KT5823 as mentioned above (Table 1, KT5823), but also by the ROS scavenger MPG (500 µM), the H_2_O_2_-decomposing enzyme catalase (500 U/ml), the cell-permeable calmodulin antagonist SKF-7171A (10 µM), and mAIP (1 µM), the myristoylated autocamtide-2 related inhibitory peptide highly selective for CaMKII, respectively (Table 1; 5–8 patches). Moreover, the shifts in the open and closed duration distributions and changes in individual time constants observed during application of zaprinast (Fig. 5A) were abolished when mAIP was coapplied (Fig. 5C; a representative cell-attached patch). Our results thus indicate that PKG activation increased the activity of Kir6.2/SUR2A channels in intact cells by altering the open and closed properties of these channels in a ROS-, calmodulin- and CaMKII-dependent manner.

### Exogenous H_2_O_2_ increased the single-channel activity of Kir6.2/SUR2A channels in cell-attached patches in a concentration-dependent manner while suppressed these channels in excised, inside-out patches

**Figure 6:**
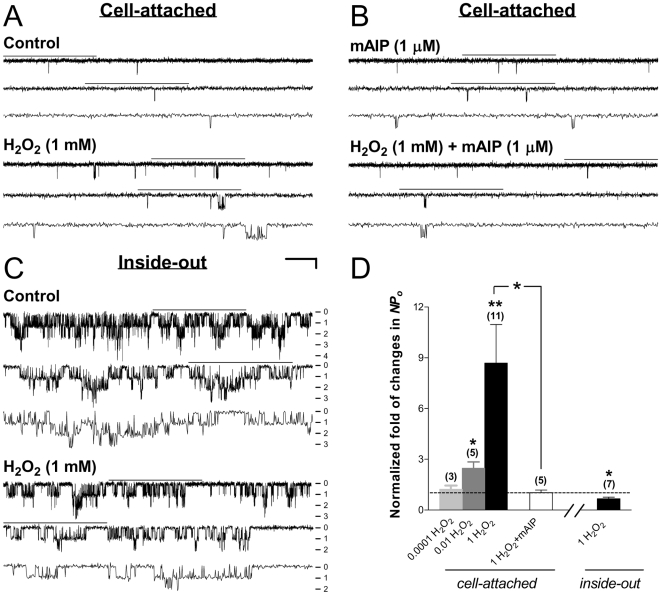
Dual effects of H_2_O_2_ on the function of cardiac-type K_ATP_ channels. Currents were obtained in the cell-attached (A,B) or inside-out (C) patch configuration from transiently transfected HEK293 cells expressing Kir6.2/SUR2A channels. (A–B) Single-channel current traces of Kir6.2/SUR2A channels in representative cell-attached patches prior to (upper panel) and during (lower panel) bath perfusion of H_2_O_2_ (1 mM) alone (A), or H_2_O_2_ plus the myristoylated CaMKII inhibitory peptide mAIP (1 µM) (B). (C) Single-channel current traces of Kir6.2/SUR2A channels from a representative inside-out patch before and during application of H_2_O_2_ (1 mM). Numbers (from 0–4) provided along the right margin of current traces indicate the increasing level of simultaneous channel opening: 0 for closed-channel, 1 for one-channel, 2 for two-channel level opening, etc. MgATP (30 µM) was included in the bath and drug solutions during inside-out recordings to prevent current rundown. The scale bars are the same as described in Fig. 1. (D) Normalized *NPo* of Kir6.2/SUR2A channels obtained during application of drugs in individual groups. The labels (*cell-attached* and *inside-out*) placed underneath the X axis depict the patch configuration in which data were obtained. *NPo* values of all groups were normalized to the corresponding control obtained prior to index drug application in individual patches as described in Fig. 1D (control taken as 1; dashed line). Data obtained from cell-attached patches treated with increasing concentrations of H_2_O_2_ (0.0001, 0.01 and 1 mM) were also displayed (first three bars from the left) to illustrate the concentration dependence of H_2_O_2_ effects. Data are presented as mean ± SEM of 3–11 patches (number of patches in individual groups provided in parentheses). Significance levels are: *, *P*<0.05; **, *P*<0.01 (two-tailed one-sample *t* tests within individual groups, or unpaired *t* tests between groups).

ROS play an important role in cell signaling. Most aspects of (oxidant) signaling have been linked to the more stable derivative, H_2_O_2_ [40]. To determine how H_2_O_2_ modulates the activity of cardiac-type K_ATP_ channels in intact HEK293 cells, we examined the changes in the single-channel currents of Kir6.2/SUR2A channels before and during bath application of H_2_O_2_ using cell-attached patch recordings. H_2_O_2_ (1 mM) increased the apparent opening frequency and the open duration of Kir6.2/SUR2A channels compared with the control obtained from the same cell-attached patch before application of H_2_O_2_ (Fig. 6A), without affecting the single-channel conductance. The averaged normalized *NPo* was 8.69±2.27 during 1 mM H_2_O_2_ application (control as 1) (Fig. 6D, 3rd bar from the left; 11 patches; *P*<0.01, one-sample *t* test), indicating that H_2_O_2_ stimulates cardiac-type K_ATP_ channels in intact HEK293 cells. We also found that H_2_O_2_ at 10 µM was still capable of increasing the normalized *NPo* of the Kir6.2/SUR2A channel (*NPo* = 2.47±0.38; Fig. 6D, 2nd bar from the left; 5 patches; *P*<0.05, one-sample *t* test) albeit the effect was weaker compared with that obtained at 1 mM; in contrast, H_2_O_2_ at 0.1 µM failed to induce any detectable change (Fig. 6D, 1st bar from the left; 3 patches; no significant change). The concentration dependence of H_2_O_2_–induced stimulation of cardiac-type K_ATP_ channels implies that the stimulatory action of H_2_O_2_ is specific.

Does H_2_O_2_ stimulation of Kir6.2/SUR2A channels in cell-attached patches (see Fig. 6A,D) result from direct or indirect modification of the channel protein? Previously we have demonstrated that direct application of H_2_O_2_ to the Kir6.2/SUR1, the neuronal-type K_ATP_, channel in inside-out patches did not increase but instead reduced the channel activity [25] even though in cell-attached patches the effect of H_2_O_2_ is stimulatory, which suggests that H_2_O_2_ achieves neuronal K_ATP_ channel stimulation by indirectly modifying the channel. To determine whether H_2_O_2_ exerts a similar action on cardiac K_ATP_ channels in the cell-free condition, we administered exogenous H_2_O_2_ by bath perfusion to inside-out membrane patches excised from transfected HEK293 cells expressing Kir6.2/SUR2A channels. MgATP (30 µM) was included in both bath and drug solutions during inside-out recordings to prevent current rundown in the cell-free condition [14], [21], [24], [25]. The K_ATP_ channel activity in inside-out patches (Fig. 6C) was much elevated compared with that in cell-attached patches (Fig. 6A), likely due to partial relief of intracellular ATP inhibition upon patch excision. We found that H_2_O_2_ decreased the Kir6.2/SUR2A channel activity in inside-out patches (Fig. 6C); the averaged normalized *NPo* was significantly reduced to 0.67±0.09 (control as 1) (Fig. 6D, rightmost bar; 7 patches; *P*<0.05, one-sample *t* test). These results indicate that in addition to the stimulatory effect observed in intact cells (see Fig. 6A,D), H_2_O_2_ suppressed Kir6.2/SUR2A channel function in the cell-free condition, possibly by direct modification of the channel or some closely associated regulatory protein(s). Because H_2_O_2_ enhanced the activity of Kir6.2/SUR2A channels only in intact cells but not in inside-out patches, the stimulatory action of H_2_O_2_ was likely indirect. Moreover, as the combined (*i.e.*, direct plus indirect) effect of H_2_O_2_ on Kir6.2/SUR2A channels in intact cells was channel activation, the stimulatory action of H_2_O_2_ appeared to be the primary effect exerted by ROS generation on these channels.

### Stimulation of Kir6.2/SUR2A channels by exogenous H_2_O_2_ in intact cells was abolished by CaMKII inhibitors

Our findings described above revealed that ROS/H_2_O_2_, calmodulin, and CaMKII were crucial for PKG stimulation of Kir6.2/SUR2A channels in intact cells (see Figs. 1, 3 and 4 and Table 1), it is therefore important to determine the relative position (or order) of ROS/H_2_O_2_ and calmodulin/CaMKII in the intracellular signaling pathway triggered by activation of PKG. If H_2_O_2_ is generated along the PKG-induced signaling pathway after the activation of CaMKII, the effect of exogenous H_2_O_2_ on Kir6.2/SUR2A channels should not be affected by functional suppression of CaMKII. To determine whether CaMKII is positioned downstream (or upstream) of ROS/H_2_O_2_ in mediating the K_ATP_ channel stimulation in intact HEK293 cells, mAIP, a myristoylated inhibitory peptide highly selective for CaMKII, was coapplied with H_2_O_2_ during cell-attached recordings of Kir6.2/SUR2A channels. We found that in the presence of mAIP (1 µM), exogenous H_2_O_2_ (1 mM) was unable to increase the single-channel activity of Kir6.2/SUR2A channels (Fig. 6B; a representative cell-attached patch); the averaged, normalized *NPo* was not different from the corresponding control in individual patches (Fig. 6D, open bar; 5 patches; one-sample *t* test) but was significantly reduced compared with the positive group H_2_O_2_ (Fig. 6D, 3rd vs. 4th bars from the left; *P*<0.05, Dunnett's multiple comparison test following one-way ANOVA). These findings were compatible with the observation that H_2_O_2_ did not directly stimulate Kir6.2/SUR2A channels (see Fig. 6A,C,D). Our data thus indicate that the activity of CaMKII was required to mediate stimulation of Kir6.2/SUR2A channels by ROS/H_2_O_2_ in intact HEK293 cells. In other words, CaMKII activation may occur downstream of ROS generation during activation of PKG to achieve functional enhancement of cardiac K_ATP_ channels.

### Effects of H_2_O_2_ on the single-channel open and closed properties of Kir6.2/SUR2A channels in intact cells

The effects of exogenous H_2_O_2_ on the single-channel open and closed properties of Kir6.2/SUR2A channels expressed in intact HEK293 cells were analyzed in cell-attached patches suitable for single-channel analysis. The normalized values of *NPo*, opening frequency, corrected mean open duration, and mean closed duration of Kir6.2/SUR2A channels obtained in intact HEK293 cells were significantly altered during H_2_O_2_ treatment (control as 1) (Table 2, H_2_O_2_; 9 patches; one-sample *t* test). The increases in the opening frequency (*P*<0.01), the corrected mean open duration (*P*<0.05), and consequently the *NPo* (*P*<0.05), plus the reduction in the mean closed duration (*P*<0.0001), provided a kinetic explanation for Kir6.2/SUR2A channel activation induced by H_2_O_2_ (Table 2, H_2_O_2_). These changes in the single-channel open and closed properties were similar to the kinetic effects exerted by zparinast on the same K_ATP_ channel isoform in intact cells (Table 1, Zaprinast). Furthermore, we found that H_2_O_2_–induced changes in the normalized single-channel open and closed properties of Kir6.2/SUR2A channels in intact HEK293 cells were completely prevented when the potent and selective CaMKII inhibitory peptide mAIP was coapplied (Table 2, mAIP). These results indicate that exogenous H_2_O_2_ stimulated Kir6.2/SUR2A channels in intact HEK293 cells by altering the gating properties of the channel in a CaMKII-dependent manner.

**Table 2:** Effects of exogenous H_2_O_2_ on the normalized single-channel open and closed properties of Kir6.2/SUR2A channels in intact HEK293 cells in the absence and presence of CaMKII inhibition.

Properties	H_2_O_2_	+mATP
Open probability	8.85±2.68*	1.02±0.15
Opening frequency	5.70±1.18**	0.83±0.11
Mean open duration	1.50±0.17*	1.27±0.16
Mean closed duration	0.25±0.06****	1.28±0.18
Number of patches	9	5

Single-channel recordings of Kir6.2/SUR2A channels in cell-attached patches obtained from transfected HEK293 cells were performed at −60 mV in symmetrical 140-mM K^+^ solutions. H_2_O_2_ (1 mM) or H_2_O_2_ (1 mM) plus the myristoylated inhibitory peptide for CaMKII mAIP (1 µM) was applied by bath perfusion using a pressure-driven perfusion system. The single-channel properties were obtained as described in Methods. All values were normalized to the corresponding controls obtained in individual patches prior to index drug application (control taken as 1), averaged and are presented as mean ± SEM. Significance levels are:

*, *P*<0.05;

**, *P*<0.01;

****, *P*<0.0001 (two-tailed one-sample *t* tests).

### Effects of PKG activation on sarcK_ATP_ channels in intact ventricular cardiomyocytes isolated from adult rabbits

**Figure 7:**
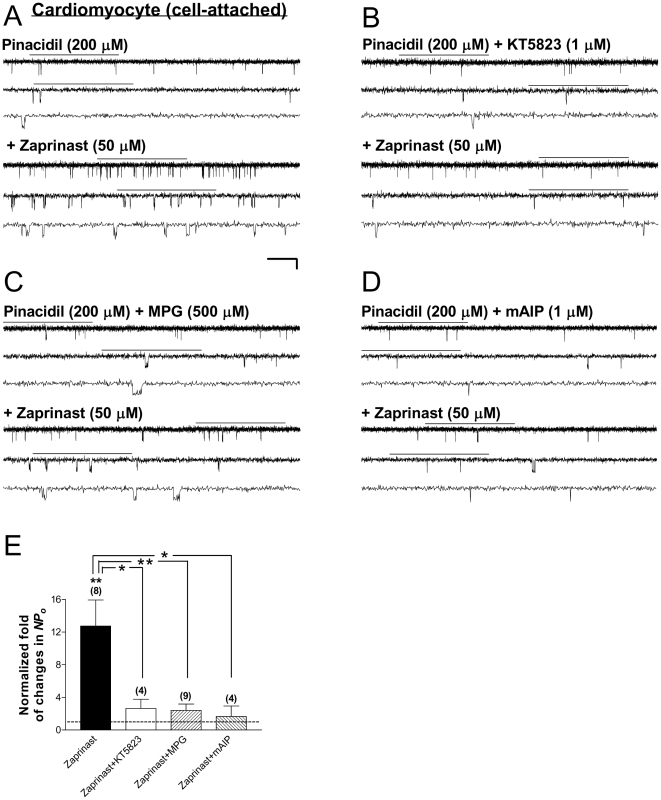
Stimulation of sarcK_ATP_ channels in ventricular cardiomyocyte by intracellular signaling triggered by activation of PKG. Recordings were performed in symmetrical high potassium (140-mM) solutions at room temperature in the cell-attached configuration and the membrane potential was clamped at −60 mV. (A–D) Single-channel current traces of the sarcK_ATP_ channel preactivated by the K_ATP_ channel opener pinacidil (200 µM) in a representative cell-attached patch prior to (upper panel) and during (lower panel) addition of the cGMP-specific PDE inhibitor zaprinast (50 µM) (A), or zaprinast (50 µM) together with one of the following: a selective PKG inhibitor KT5823 (1 µM) (B), a membrane-permeable ROS scavenger MPG (500 µM) (C), or the myristoylated autocamtide-2 related inhibitory peptide for CaMKII (mAIP; 1 µM) (D). Cells were pretreated with respective inhibitors for at least 15 min at room temperature before recordings were started. Scale bars are the same as described in Fig. 1. Downward deflections represent openings from closed states. (E) The averaged normalized *NPo* values of sarcK_ATP_ channels obtained during application of drugs in individual groups of cell-attached patches. *NPo* values were normalized to the corresponding control in pinacidil with or without inhibitors (control taken as 1; dashed line). Data are presented as mean ± SEM of 4–9 patches. Significance levels are: *, *P*<0.05; **, *P*<0.01 (two-tailed one-sample *t* tests within individual groups, or Dunnett's multiple comparison tests between groups).

Finally, to confirm the relevance of the findings obtained from recombinant cardiac-type K_ATP_ channels to the native system, we examined the effect of PKG activators on the activity of sarcK_ATP_ channels in cell-attached patches obtained from intact ventricular cardiomyocytes acutely isolated from adult rabbits. The K_ATP_ channel opener pinacidil (200 µM) was applied first to induce sufficient baseline channel activity in the control condition for subsequent pairwise comparisons. In the continuous presence of pinacidil (200 µM), bath perfusion of the cGMP-seletive PDE inhibitor zparinast (50 µM) effectively potentiated the single-channel activity of sarcK_ATP_ channels (Fig. 7A); the normalized *NPo* was 12.74±3.21 (control in pinacidil taken as 1) (Fig. 7E, filled bar; 8 patches; *P*<0.01, two-tailed one-sample *t* test). In contrast, zaprinast was incapable of further enhancing pinacidil-induced sarcK_ATP_ channel activity when KT5823, a selective PKG inhibitor, was coapplied (Fig. 7B,E, open bar; 4 patches; no significant change), yielding significant ablation of PKG-induced channel stimulation (*NPo* = 2.64±1.13; Fig. 7E, filled vs. open bars; *P*<0.05, Dunnett's multiple comparison test following one-way ANOVA). These results indicate that zaprinast stimulated sarcK_ATP_ channels in intact ventricular cardiomyocytes via activation of PKG, which was in line with findings obtained from recombinant cardiac-type K_ATP_ channels in HEK293 cells (see Fig. 1).

### Effects of ROS scavenging and inhibition of CaMKII on PKG stimulation of sarcK_ATP_ channel activity in intact ventricular cardiomyocytes

Because PKG did not activate Kir6.2/SUR2A channels in excised, inside-out patches (see Fig. 2), it is less likely that the channel subunits serve as direct targets of PKG for channel stimulation. The key question to be addressed next is whether PKG activation stimulates sarcK_ATP_ channels in cardiomyocytes via intracellular signaling, as our recombinant channel data have implied (see Figs. 1–6), or by phosphorylation of some regulatory protein associated closely with the channel in the plasma membrane, as indirectly inferred from the findings made by Han *et al*. [26], [27]. To determine the role of ROS in mediating the stimulatory effect of PKG on sarcK_ATP_ channels, single-channel recording experiments were performed in cell-attached patches obtained from rabbit ventricular cardiomyocytes. SarcK_ATP_ channels were preactivated with pinacidil (200 µM) to induce basal activity sufficient for subsequent pairwise comparisons. We found that coapplication of zaprinast (50 µM) and the ROS scavenger MPG (500 µM) in the continuous presence of pinacidil did not result in an increase in the single-channel activity of sarcK_ATP_ channels (Fig. 7C); the normalized *NPo* was 2.40±0.78 (Fig. 7E, 3rd bar from the left; 9 patches; no significant change, one-sample *t* test), revealing a significant nullification of the positive zaprinast effect by MPG (Fig. 7E, 1st vs. 3rd bars; *P*<0.01, Dunnett's multiple comparison test following one-way ANOVA). Furthermore, to determine whether CaMKII activation is required for PKG stimulation of sarcK_ATP_ channels in intact ventricular cardiomyocytes, PKG activators zaprinast was applied by bath perfusion together with mAIP, the potent and highly selective inhibitory peptide for CaMKII. Activation of PKG by zaprinast (50 µM) failed to increase the single-channel activity of sarcK_ATP_ channels preactivated by pinacidil (200 µM) when mAIP (1 µM) was coadministered (*NPo* = 1.66±1.28; Fig. 7D,E, 4th bar from the left; 4 patches; no significant change), and PKG's stimulatory effect was significantly ablated by inhibition of CaMKII (Fig. 7E, 1st vs. 4th bars; *P*<0.05, Dunnett's multiple comparison test). The results obtained from these two experimental groups performed in intact ventricular cardiomyocytes were consistent with, and thereby confirmed, the findings made in intact HEK293 cells expressing recombinant Kir6.2/SUR2A channels (see Figs. 3A,C and 4B,C), indicating that the stimulatory action of PKG activation on sarcK_ATP_ channels in intact ventricular cardiomyocytes were ROS- and CaMKII-dependent.

## Discussion

Vital in the adaptive response to (patho)physiological stress, K_ATP_ channels serve a homeostatic role ranging from glucose regulation to cardioprotection [41]. The K_ATP_ channel is important for cardiac function; indeed, genetic disruption of the pore-forming subunit that comprises cardiac K_ATP_ channels renders the knockout mice less tolerant to different types of stress, resulting in abnormal cytosolic calcium handling, susceptibility to developing acute cardiac failure, and sudden cardiac death [42], [43]. In the present study, we demonstrated that PKG exerted bidirectional modulation of cardiac-type K_ATP_ (*i.e.*, Kir6.2/SUR2A) channel function, the major effect of which was an indirect, stimulatory action resulting from intracellular signaling mediated by ROS (in particular H_2_O_2_), calmodulin, and CaMKII; on the other hand, direct PKG phosphorylation of the channel was unlikely involved in channel stimulation but instead may cause moderate channel inhibition. We also defined the kinetic basis on which PKG signaling enhanced the function of Kir6.2/SUR2A channels in intact cells, and provided novel evidence that enhancement of cardiac-type K_ATP_ channels by ROS/H_2_O_2_ relied on activation of CaMKII. And lastly, we examined the effect of PKG activation and the roles played by key signaling components ROS and CaMKII in mediating the modulatory effect of PKG activation on sarcK_ATP_ channels in ventricular cardiomyocytes to confirm the physiological relevance of our findings obtained from transfected HEK293 cells.

### Bidirectional regulation of the cardiac-type K_ATP_ channel by PKG-mediated phosphorylation: direct and indirect effects

The cGMP/PKG signaling mechanism is involved in the regulation of smooth muscle relaxation, learning and memory, cell division, and cardioprotection [44], [45]. In the present study, accumulation of intracellular cGMP by application of the membrane-permeable cGMP analog 8-Br-cGMP (Fig. 1A,D) or the membrane-permeable, cGMP-specific PDE inhibitor zaprinast (Figs. 1B,D and S1; Table 1) resulted in cardiac-type K_ATP_ (*i.e.*, Kir6.2/SUR2A) channel activation in intact HEK293 cells. Importantly, the stimulatory effect of zaprinast was concentration-dependent (0.05–50 µM; Fig. 1D), suggesting that the stimulatory action of zparinast on the K_ATP_ channel in intact cells is specific. Further, the stimulatory effects of zaprinast on the single-channel activity of Kir6.2/SUR2A channels were significantly abolished by KT5823, a selective, membrane-permeable PKG inhibitor (Fig. 1C,D; Table 1, Zaprinast and Zaprinast+KT5823). KT5823 at the concentration of 1 µM should effectively and selectively inhibit PKG, because its IC_50_ is 0.23 µM for PKG but is much higher (10 µM) for PKA. In line with this prediction, we have demonstrated that the action of the PKG activator zaprinast on the neuronal-type K_ATP_ channel is abolished by KT5823 at 1 µM whereas unaffected by the selective PKA inhibitor KT5720 [25]. The stimulatory effect of PKG activation on cardiac K_ATP_ channels was confirmed in intact ventricular cardiomyocytes (Fig. 7A,B,E; Table S1, Zaprinast). Our new data (Figs. 1 and 7A,B,E; Tables 1 and S1) thus suggest that activation of PKG by accumulation of intracellular cGMP positively modulates the function of cardiac K_ATP_ channels in intact cells.

In addition to the stimulatory action of PKG on the activity of Kir6.2/SUR2A channels in intact cells, we also demonstrated that direct application of either the catalytic subunit of PKG (Fig. 2A) or the PKG holoenzyme (Fig. 2C) to the cytoplasmic surface of inside-out patches excised from HEK293 cells did not stimulate but reduced Kir6.2/SUR2A channel function (Fig. 2A,C,E). The inhibitory action of PKG was abolished by heat inactivation of the enzyme (Fig. 2B,D), implying a specific enzyme effect. Our results thus suggest that PKG phosphorylation of the cardiac K_ATP_ channel or some closed associated regulatory protein in the plasma membrane renders channel inhibition, an effect likely masked by the predominating, stimulatory effect of PKG in intact cells (Fig. 1).

However, our findings on the inhibitory effect (*i.e.*, channel suppression) of PKG on Kir6.2/SUR2A channels in inside-out patches (Fig. 2) seem to be in contradiction to those reported by Han *et al.* [26], in which a positive effect (*i.e.*, channel activation) was induced by brief application of PKG to sarcK_ATP_ channels in inside-out patches excised from native cardiomyocytes. Thus far we were unable to reproduce the stimulatory PKG effect in inside-out patches obtained from rabbit ventricular myocytes (Lin and Chai, unpublished data), the same cell model used by Han *et al.* [26], and we suspect that the discrepancy in experimental findings may result from, among others, some difference in the drug application protocol employed. For instance, instead of simultaneously administering enzymes together with their coactivators (MgATP/cGMP or cGMP) [26], [27], in our inside-out patch recording experiments we applied individual solutions containing the coactivators or coactivator plus PKG in an incremental manner. In other words, we did not switch to a perfusion solution containing additional chemicals or reagents until the channel activity in the present solution has become stable, and typically we recorded in each drug solution for 6–12 min. We consider this routine more suitable for detecting and comparing the steady-state response of channels to pharmacological treatments. Our findings that the steady-state response of Kir6.2/SUR2A channels in inside-out patches to direct application of PKG (either the holoenzyme or the catalytic subunit) was a reduction in channel function (see Fig. 2) instead of channel stimulation as seen in the cell-attached patches (see Fig. 1) suggest the following. First, PKG does not directly phosphorylate the cardiac K_ATP_ channel protein to induce channel activation observed in intact cells (Figs. 1 and 7). Second, PKG may directly phosphorylate the cardiac K_ATP_ channel or some closely associated regulatory protein to render modest to moderate suppression of the channel function (Fig. 2). On the other hand, because proper cGMP controls and time controls were not secured prior to the administration of exogeneous PKG, it is not clear whether the stimulatory effect on sarcK_ATP_ channel activity in inside-out patches excised from ventricular cardiomyocytes during brief cGMP/PKG application reported by Han *et al.* [26] is a PKG-specific action. Nonetheless, our cloned channel data of the present study (Figs. 1 and 2) provide unambiguous support to suggest that the stimulatory action of PKG on cardiac K_ATP_ channels observed in intact cells does not result from direct PKG phosphorylation of the channel; in other words, some indirect mechanism is responsible for cardiac K_ATP_ channel stimulation by PKG (see next).

Together, our findings obtained from cell-attached (Fig. 1; Table 1) and inside-out (Fig. 2) patches from HEK293 cells expressing cardiac-type K_ATP_ channels and from ventricular cardiomyocytes (Fig. 7A,B,E; Table S1) suggest that PKG exerts bidirectional regulation of cardiac K_ATP_ channel function; the stimulatory action of PKG is likely dependent on some cytosolic, intermediate messenger(s) whereas the inhibitory action of PKG may be attributed to direct PKG phosphorylation of the channel or some closely associated regulatory protein(s). We further suggest that the stimulatory action of PKG predominates over its inhibitory action, as the latter was completely masked in intact cells (Figs. 1 and 7; Tables 1 and S1). Hence, the stimulatory action of PKG may represent the primary effect exerted by PKG phosphorylation on cardiac K_ATP_ channel modulation. These results were reminiscent of the bidirectional modulation of the Kir6.2/SUR1 (*i.e.*, neuronal-type K_ATP_) channel function by PKG we have previously demonstrated in two different cell models [24], and therefore implicate that PKG may modulate the function of K_ATP_ channels in the heart and the brain through some common mechanism.

### ROS and particularly H_2_O_2_ mediate PKG-induced stimulation of cardiac K_ATP_ channels in intact cells

ROS are generated by all aerobic cells, and most endogenously produced ROS are derived from mitochondrial respiration [46], [47]. ROS have been shown to contribute to the cardioprotection afforded by ischemic preconditioning [48], [49]. Among all ROS, H_2_O_2_ is an attractive candidate for cell signaling, because compared with other ROS it is relatively stable and long-lived, and its neutral ionic state allows it to exit the mitochondria easily [50]. In this study the stimulation of Kir6.2/SUR2A channels by PKG activation in intact HEK293 cells was abolished by ROS scavenging (Fig. 3A,C; Table 1). Moreover, the stimulatory effects of PKG activation were abrogated by catalase, an enzyme that decomposes H_2_O_2_ (Fig. 3B,C; Table 1). Similarly, PKG stimulation of sarcK_ATP_ channels in intact ventricular cardiomyocytes was also prevented in the presence of ROS scavengers (Fig. 7C,E; Table S1, MPG). Our findings (Figs. 3 and 7C,E; Tables 1 and S1) thus suggest that the PKG-induced stimulation of cardiac K_ATP_ channels is mediated by ROS/H_2_O_2_ signaling. We have previously shown that ROS are indispensible for PKG stimulation of the neuronal-type K_ATP_ channel [25]. It is conceivable that ROS may function as a critical signal in PKG signaling to modulate K_ATP_ channels in different tissues.

### Calmodulin and CaMKII are required for cardiac K_ATP_ channel stimulation by PKG in intact cells

Ca^2+^/calmodulin-dependent kinases (CaMKs) influence processes as diverse as gene transcription, cell survival, apoptosis, cytoskeletal re-organization and learning and memory. CaMKII is the CaMK isoform predominantly found in the heart [51]. We have previously demonstrated that intracellular calcium and calmodulin mediate the stimulatory effect of PKG signaling on neuronal-type K_ATP_ channels [25]. Results obtained from the present study further suggest that PKG enhances cardiac K_ATP_ channel function via activation of the Ca^2+^-binding protein calmodulin and CaMKII, because not only PKG stimulation of the Kir6.2/SUR2A channel in intact HEK293 cells was completely nullified by SKF-7171A (a selective calmodulin antagonist) (Fig. 4A,C; Table 1) and mAIP (a myristoylated autocamtide-2 related inhibitory peptide for CaMKII) (Fig. 4B,C; Table 1), PKG stimulation of sarcK_ATP_ channels in ventricular cardiomyocytes was also ablated by inhibition of CaMKII with mAIP (Fig. 7D,E). It has been suggested that increased short-term CaMKII activity may serve as beneficial negative feedback for calcium on repolarization of cardiomyocyte membranes [39]. Putative substrates for CaMKII include proteins involved in regulating Ca^2+^ storage and release, transcription factors, and ion channels [37]. Further study is required to elucidate how CaMKII modulates the function of cardiac K_ATP_ channels.

### PKG signaling modifies the single-channel open and closed properties of cardiac-type K_ATP_ channels to achieve channel activation

Based on the single-channel analysis of open- and closed-duration distributions of Kir6.2/SUR2A channels in intact HEK293 cells, we suggest that the cardiac-type K_ATP_ channel exhibits at least two open states and three closed states (Fig. 5). The effects of the PKG activator zaprinast on the open- and closed-duration distributions of Kir6.2/SUR2A channels (Fig. 5A) imply that zaprinast stimulates cardiac K_ATP_ channels by destabilizing the longest closed conformation whereas stabilizing the long open conformation. Moreover, zaprinast also facilitated the closed-to-open transitions (*i.e.*, opening frequency) of the channel and elevated the *NPo* (Table 1). All these changes induced by zaprinast in the single-channel properties of Kir6.2/SUR2A channels were sensitive to the PKG inhibitor and therefore may constitute the kinetic mechanism responsible for PKG stimulation of cardiac K_ATP_ channels (Fig. 5B; Table 1). These kinetic changes induced by zaprinast were also abolished by scavenging of ROS, enzymatic decomposition of H_2_O_2_, inhibition of calmodulin, and blockade of CaMKII activation (Fig. 5C; Table 1), suggesting the involvement of ROS, especially H_2_O_2_ and related species, calmodulin, and CaMKII in mediating sGC/PKG stimulation of cardiac K_ATP_ channels through altering the open and closed properties of the channel. These results provide kinetic insights into the functional modulation of cardiac-type K_ATP_ channels by an intracellular signaling mechanism triggered by PKG.

### H_2_O_2_ indirectly stimulates cardiac-type K_ATP_ channels in intact cells

Our findings (see Figs. 3 and 7C,E) of the present study imply a permissive role of ROS, especially H_2_O_2_, in mediating cardiac K_ATP_ channel stimulation downstream of PKG activation in intact cells. Importantly, in the present study we provide direct evidence that H_2_O_2_ concentration-dependently stimulated the single-channel activity of Kir6.2/SUR2A channels in intact HEK293 cells (Fig. 6A,D; Table 2), suggesting that the cardiac K_ATP_ channel is positively modulated by H_2_O_2_ in intact cells. H_2_O_2_ has been suggested to regulate K_ATP_ channel activity or K_ATP_ channel-related cellular function in several cell types. For example, H_2_O_2_ causes sulfonylurea-sensitive hyperpolarization and suppression of insulin release in pancreatic β-cells [52], and mediates glutamate-dependent inhibition of dopamine release from striatum by activating K_ATP_ channels [53], [54]. H_2_O_2_ has also been shown to regulate K_ATP_ channel activity in ventricular cardiomyocytes [55]–[57]. However, the activity of Kir6.2/SUR2A channels in inside-out patches was suppressed rather than stimulated by H_2_O_2_ (Fig. 6C,D). It thus appeared that the direct action of ROS/H_2_O_2_, possibly by oxidizing the channel protein or some closely associated regulatory protein, is inhibitory, which rules out direct oxidation of redox-sensitive sites on the Kir6.2/SUR2A channel as a potential cause responsible for ROS/H_2_O_2_-induced channel stimulation in intact cells (Fig. 6A). Indeed, strong oxidants or sulfhydryl oxidizing agents cause K_ATP_ channel closure in skeletal muscle cells [58], pancreatic β-cells [59] and cardiac cells [60]. Together, our findings on recombinant cardiac-type K_ATP_ channels support that H_2_O_2_ stimulates cardiac K_ATP_ channels via an indirect mechanism rather than by direct chemical modification of the channel. The H_2_O_2_-induced stimulation of Kir6.2/SUR2A channels in intact cells (Fig. 6A,D; Table 2, H_2_O_2_) likely represents a summated outcome of the dual action of H_2_O_2_, in which the channel stimulation predominates over and masks the inhibition. The bidirectional regulation of cardiac-type K_ATP_ channel function by H_2_O_2_ (Fig. 6) was reminiscent of the H_2_O_2_ effects on neuronal-type K_ATP_ channels we have recently demonstrated [25], suggestive of a common mechanism underlying ROS/H_2_O_2_ modulation of K_ATP_ channels in different tissues.

### Activation of CaMKII mediates H_2_O_2_ stimulation of cardiac K_ATP_ channels in intact cells

In the present study, suppression of CaMKII activity with a potent and highly selective peptide inhibitor mAIP significantly abolished the stimulatory effect of H_2_O_2_ on Kir6.2/SUR2A channels in intact HEK293 cells (Figs. 6B,D; Table 2), suggesting that CaMKII serves as a downstream signaling component to mediate stimulatory actions of H_2_O_2_ (Fig. 6A,D) and PKG (Figs. 1) on cardiac K_ATP_ channels. These findings were also compatible with the observation that H_2_O_2_ did not stimulate Kir6.2/SUR2A channels directly in intact cells (see Fig. 6A,C,D). The crucial role of CaMKII in mediating H_2_O_2_-induced changes in the kinetic properties of cardiac-type K_ATP_ channels, which in turn resulted in enhanced channel activity, was also supported by our data on single-channel open and closed properties (Table 2). H_2_O_2_ may activate CaMKII by increasing the calcium permeability from intracellular stores [61] and by activating calmodulin [25], and consequently stimulates K_ATP_ channels in the plasma membrane. Although recent evidence indicates that direct oxidation of CaMKII by ROS (generated downstream of angiotensin II) may sustain CaMKII activity in the absence of Ca^2+^/calmodulin [62], our findings that the stimulatory action of H_2_O_2_ on Kir6.2/SUR2A channels was completely abolished by suppression of calmodulin (Fig. S2) implicate an involvement of the Ca^2+^/calmodulin mechanism in rendering ROS activation of CaMKII. Moreover, our recent study has provided evidence for a role of intracellular Ca^2+^ in mediating PKG stimulation of neuronal K_ATP_ channels in intact cells (Chai and Lin, 2010), which is in line with the current working model for a Ca^2+^/calmodulin-dependent activation of CaMKII downstream of PKG and ROS signaling.

In conclusion, here we report for the first time that PKG bidirectionally modulates cardiac K_ATP_ channels; PKG stimulates cardiac K_ATP_ channels via an intracellular signaling mechanism consisting of ROS (particularly H_2_O_2_), calmodulin, and CaMKII, whereas inhibits the channel likely by direct PKG phosphorylation of the channel or some closely associated regulatory protein. Mechanistic understanding of K_ATP_ channel regulation may provide insights into the development of strategies for the management of cardiovascular injury. It is noteworthy that K_ATP_ channels, ROS, and cGMP-selective phosphodiesterase V (PDE V) inhibitors have been implicated in cardiac protection/tolerance against ischemic injury. Hence, this novel cGMP/PKG/ROS/calmodulin/CaMKII/K_ATP_ signaling pathway may regulate cardiomyocyte excitability and contribute to endogenous cardioprotective mechanisms. Further, this novel pathway may represent a common mechanism for K_ATP_ channel modulation in tissues including the heart and the brain and thus will be of broad physiological importance.

## Supporting Information

Figure S1 **Effects of PKG activation on the absolute open probability of cardiac-type K_ATP_ channels in individual cell-attached patches.** Recombinant Kir6.2/SUR2A channels were expressed in HEK293 cells by transient transfection. The cGMP-dependent PDE inhibitor zaprinast was administered by bath perfusion to activate PKG. Colored lines depict pairs of the absolute *NPo* data obtained from the same cell-attached patches before and during application of zaprinast (50 µM). The average *NPo* was 0.04±0.01 in the control condition, which ranged from 0.01 to 0.1%, and was 0.48±0.16 during application of zaprinast, which ranged from 0.04 to 1.94%. The absolute *NPo* values of Kir6.2/SUR2A channels in individual patches were significantly enhanced by the PKG activator zaprinast (open diamonds) from their corresponding controls (open triangles) (*P*<0.05, two-tailed paired *t* test). The median *NPo* values (depicted as the horizontal grey bars) were 0.025 and 0.27 under control and zaprinast-treated conditions, respectively, which also exhibit an increase of around 10-fold. The distribution and changes of the absolute *NPo* before and during zaprinast treatment indicate that PKG activation significantly increased the (absolute) single-channel activity of Kir6.2/SUR2A channels in intact HEK293 cells.
(TIF)

Figure S2 **Role of calmodulin in mediating the stimulatory effect of H_2_O_2_ on Kir6.2/SUR2A channels in intact HEK293 cells.** Recombinant Kir6.2/SUR2A channels were expressed in HEK293 cells by transient transfection. Cell-attached patch recordings were performed as described in Fig. 1 of the main text. (**A**) Single-channel current traces of the Kir6.2/SUR2A channel obtained from a representative cell-attached patch prior to (upper panel) and during (lower panel) application of H_2_O_2_ (1 mM) in the continuous presence of the irreversible calmodulin antagonist SKF-7171A (10 µM), following a 15-min pretreatment with SKF-7171A (10 µM). Scale bars are the same as described in Fig. 1. (**B**) The averaged normalized *NPo* of Kir6.2/SUR2A channels in cell-attached patches obtained during application of H_2_O_2_ in the absence (filled bar) or presence (open bar) of SKF-7171A. *NPo* values were normalized to the corresponding controls (taken as 1; dashed line) obtained prior to index drug application in individual patches. The H_2_O_2_ data (1 mM; filled bar) are the same as presented in Fig. 6D, and are included here for comparison purpose. Data are presented as mean ± SEM of 3–11 patches. Significance levels are: *, *P*<0.05; **, *P*<0.01 (two-tailed one-sample *t* tests within individual groups, or unpaired *t* tests between groups). In the presence of SKF-7171A, H_2_O_2_ did not enhance the normalized *NPo* of Kir6.2/SUR2A channels in cell-attached patches; the stimulatory effect of H_2_O_2_ was completely abrogated by SKF-7171A (*P*<0.05). These results indicate that the activity of calmodulin was necessary for H_2_O_2_ stimulation of cardiac-type K_ATP_ channels in intact cells, implying the involvement of the Ca^2+^/calmodulin pathway in mediating activation of CaMKII by ROS/H_2_O_2_. Furthermore, the dependence of H_2_O_2_ effects on the activities of calmodulin (this figure) and CaMKII (Fig. 6B,D; Table 2) was in line with the data obtained from the PKG activator group (Figs. 1, 4 and 7; Table 1) and supports our hypothesis that PKG activation enhances cardiac K_ATP_ channel function via ROS generation and subsequent activation of calmodulin/CaMKII signaling in intact cells.
(TIF)

Table S1 **Effects of zaprinast on the normalized single-channel open and closed properties of sarcK_ATP_ channels in intact rabbit ventricular cardiomyocyres.** Single-channel recordings of sarcK_ATP_ channels in cell-attached patches obtained from rabbit ventricular cardiomyocytes were performed at −60 mV in symmetrical 140-mM K^+^ solutions. The baseline K_ATP_ activity was first induced by pinacidil (200 µM) before addition of zaprinast (50 µM) or zaprinast plus the ROS scavenger MPG (500 µM). All drugs were applied by bath perfusion using a pressure-driven system. The single-channel properties were obtained as described in Methods. All values were normalized to the corresponding controls (pinacidil alone) obtained in individual patches prior to index drug application (control taken as 1), averaged and are presented as mean ± SEM. Significance levels are: *, *P*<0.05; ***, *P*<0.001; ****, *P*<0.0001 (two-tailed one-sample *t* tests).
(DOC)
