# Supplementary figures and images for: Activation of cGMP-Dependent Protein Kinase Stimulates Cardiac ATP-Sensitive Potassium Channels via a ROS/Calmodulin/CaMKII Signaling Cascade
Source: PLoS One. 2011 Mar 29;6(3):e18191. doi: 10.1371/journal.pone.0018191 (PMC3066208; doi:10.1371/journal.pone.0018191)

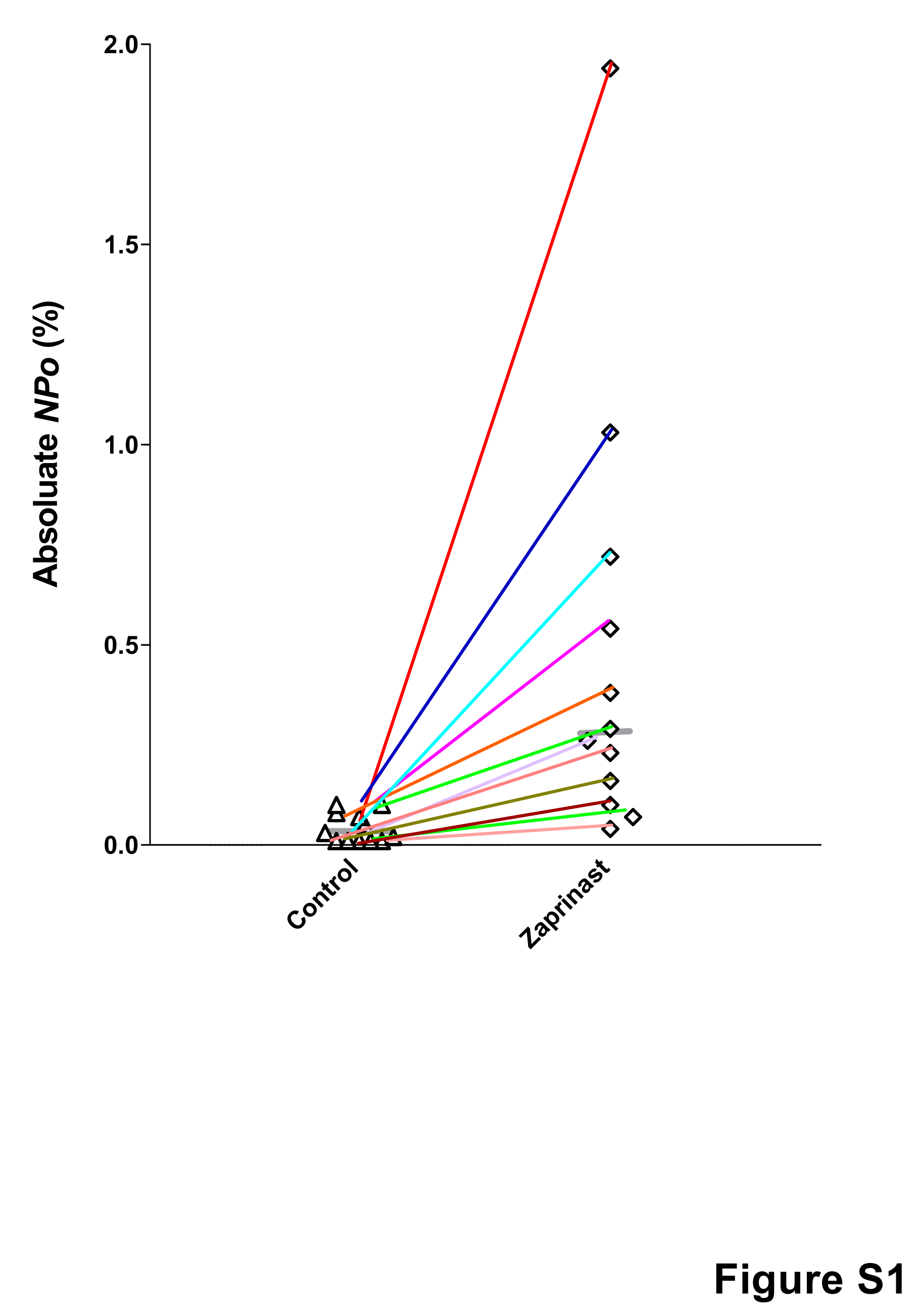

Supplement: Figure S1 — Effects of PKG activation on the absolute open probability of cardiac-type KATP channels in individual cell-attached patches. Recombinant Kir6.2/SUR2A channels were expressed in HEK293 cells by transient transfection. The cGMP-dependent PDE inhibitor zaprinast was administered by bath perfusion to activate PKG. Colored lines depict pairs of the absolute NPo data obtained from the same cell-attached patches before and during application of zaprinast (50 µM). The average NPo was 0.04±0.01 in the control condition, which ranged from 0.01 to 0.1%, and was 0.48±0.16 during application of zaprinast, which ranged from 0.04 to 1.94%. The absolute NPo values of Kir6.2/SUR2A channels in individual patches were significantly enhanced by the PKG activator zaprinast (open diamonds) from their corresponding controls (open triangles) (P<0.05, two-tailed paired t test). The median NPo values (depicted as the horizontal grey bars) were 0.025 and 0.27 under control and zaprinast-treated conditions, respectively, which also exhibit an increase of around 10-fold. The distribution and changes of the absolute NPo before and during zaprinast treatment indicate that PKG activation significantly increased the (absolute) single-channel activity of Kir6.2/SUR2A channels in intact HEK293 cells. (TIF) [file pone.0018191.s001.tif]

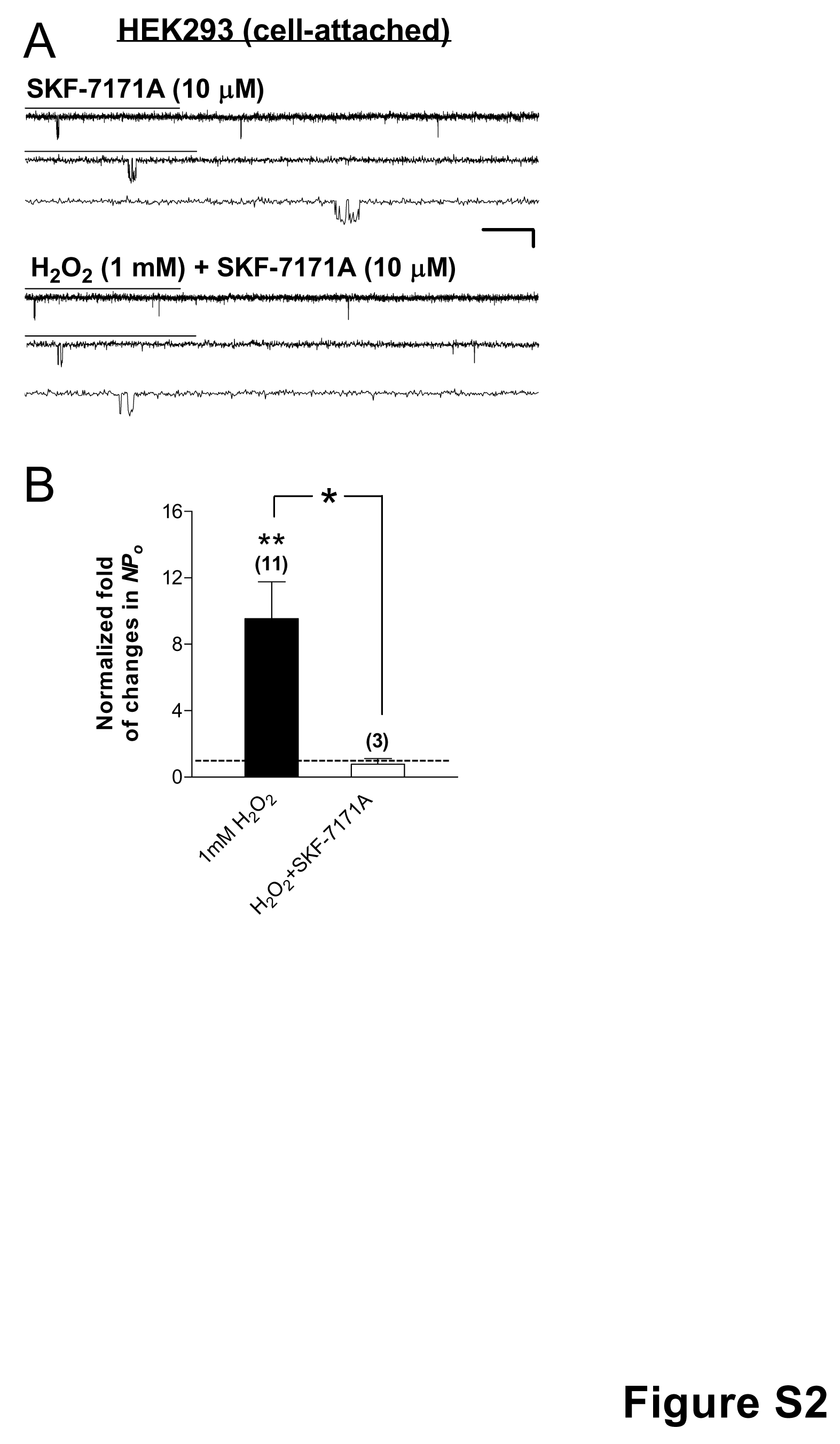

Supplement: Figure S2 — Role of calmodulin in mediating the stimulatory effect of H2O2 on Kir6.2/SUR2A channels in intact HEK293 cells. Recombinant Kir6.2/SUR2A channels were expressed in HEK293 cells by transient transfection. Cell-attached patch recordings were performed as described in Fig. 1 of the main text. (A) Single-channel current traces of the Kir6.2/SUR2A channel obtained from a representative cell-attached patch prior to (upper panel) and during (lower panel) application of H2O2 (1 mM) in the continuous presence of the irreversible calmodulin antagonist SKF-7171A (10 µM), following a 15-min pretreatment with SKF-7171A (10 µM). Scale bars are the same as described in Fig. 1. (B) The averaged normalized NPo of Kir6.2/SUR2A channels in cell-attached patches obtained during application of H2O2 in the absence (filled bar) or presence (open bar) of SKF-7171A. NPo values were normalized to the corresponding controls (taken as 1; dashed line) obtained prior to index drug application in individual patches. The H2O2 data (1 mM; filled bar) are the same as presented in Fig. 6D, and are included here for comparison purpose. Data are presented as mean ± SEM of 3–11 patches. Significance levels are: *, P<0.05; **, P<0.01 (two-tailed one-sample t tests within individual groups, or unpaired t tests between groups). In the presence of SKF-7171A, H2O2 did not enhance the normalized NPo of Kir6.2/SUR2A channels in cell-attached patches; the stimulatory effect of H2O2 was completely abrogated by SKF-7171A (P<0.05). These results indicate that the activity of calmodulin was necessary for H2O2 stimulation of cardiac-type KATP channels in intact cells, implying the involvement of the Ca2+/calmodulin pathway in mediating activation of CaMKII by ROS/H2O2. Furthermore, the dependence of H2O2 effects on the activities of calmodulin (this figure) and CaMKII (Fig. 6B,D; Table 2) was in line with the data obtained from the PKG activator group (Figs. 1, 4 and 7; Table 1) and supports our hypothesis that PKG [file pone.0018191.s002.tif]
