# Supplementary material for: Activation of cGMP-Dependent Protein Kinase Stimulates Cardiac ATP-Sensitive Potassium Channels via a ROS/Calmodulin/CaMKII Signaling Cascade
Source: PLoS One. 2011 Mar 29;6(3):e18191. doi: 10.1371/journal.pone.0018191 (PMC3066208; doi:10.1371/journal.pone.0018191)
Supplement: Table S1 — Effects of zaprinast on the normalized single-channel open and closed properties of sarcKATP channels in intact rabbit ventricular cardiomyocyres. Single-channel recordings of sarcKATP channels in cell-attached patches obtained from rabbit ventricular cardiomyocytes were performed at −60 mV in symmetrical 140-mM K+ solutions. The baseline KATP activity was first induced by pinacidil (200 µM) before addition of zaprinast (50 µM) or zaprinast plus the ROS scavenger MPG (500 µM). All drugs were applied by bath perfusion using a pressure-driven system. The single-channel properties were obtained as described in Methods. All values were normalized to the corresponding controls (pinacidil alone) obtained in individual patches prior to index drug application (control taken as 1), averaged and are presented as mean ± SEM. Significance levels are: *, P<0.05; ***, P<0.001; ****, P<0.0001 (two-tailed one-sample t tests). (DOC) [file pone.0018191.s003.doc]

| **Table S1.** *Effects of zaprinast on the normalized single-channel open and closed properties of sarcK_ATP_ channels in intact rabbit ventricular cardiomyocyres.* | | |
| --- | --- | --- |
| Properties | Zaprinast | + MPG |
| Open probability | 14.25 ± 3.26^***^ | 2.70 ± 0.99 |
| Opening frequency | 11.30 ± 2.47^***^ | 1.97 ± 0.55 |
| Mean open duration | 1.38 ± 0.16^*^ | 1.20 ± 0.12 |
| Mean closed duration | 0.13 ± 0.04^****^ | 1.09 ± 0.56 |
| Number of patches | 7 | 7 |
|  | | |
|  | | |
